# Astrocytic TCF7L2 Impacts Brain Osmoregulation and Restricts Neuronal Excitability

**DOI:** 10.1002/glia.70103

**Published:** 2025-12-05

**Authors:** Mariusz Popek, Krzysztof Goryca, Dorota Adamska, Joanna Urban‐Ciećko, Katarzyna Hryniewiecka, Marcin Lipiec, Tomasz Grzegorz Krawczyk, Kamil Rafalko, Alicja Ławicka, Shane A. Liddelow, Lukasz Mateusz Szewczyk

**Affiliations:** ^1^ Department of Neurotoxicology Mossakowski Medical Research Institute, Polish Academy of Sciences Warsaw Poland; ^2^ Genomics Core Facility, Centre of New Technologies University of Warsaw Warsaw Poland; ^3^ Laboratory of Electrophysiology Nencki Institute of Experimental Biology, Polish Academy of Sciences Warsaw Poland; ^4^ Laboratory of Molecular Neurobiology, Centre of New Technologies University of Warsaw Warsaw Poland; ^5^ Laboratory of Emotions Neurobiology, BRAINCITY – Center of Excellence for Neural Plasticity and Brain Disorders, Nencki Institute of Experimental Biology, Polish Academy of Sciences Warsaw Poland; ^6^ SoftwareMill Warsaw Poland; ^7^ Independent Researcher Warsaw Poland; ^8^ Institute for Translational Neuroscience NYU Grossman School of Medicine New York New York USA; ^9^ Parekh Center for Interdisciplinary Neurology NYU Grossman School of Medicine New York New York USA; ^10^ Department of Neuroscience NYU Grossman School of Medicine New York New York USA; ^11^ Department of Ophthalmology NYU Grossman School of Medicine New York New York USA

**Keywords:** astrocyte, neuron, TCF7L2, transcription factor, Wnt/β‐catenin

## Abstract

Astrocytes differentiate and mature during postnatal development, but the molecular mechanisms linking their maturation to neuronal function remain unclear. We investigated the role of Wnt/β‐catenin signaling and its effector, the transcription factor TCF7L2, in postnatal astrocytes using single‐nucleus RNA sequencing, imaging, morphometric analysis, microdialysis, and electrophysiology in *Tcf7l2* conditional knockout (cKO) mice. Loss of *Tcf7l2* caused widespread transcriptional dysregulation in astrocytes, particularly in genes related to amino acid and ion transport, as well as membrane potential regulation. These mice showed disrupted amino acid homeostasis, astrocyte swelling, and impaired extracellular potassium clearance in the somatosensory cortex. These astrocytic changes were accompanied by altered gene expression in cortical pyramidal neurons, reduced excitability, and a hyperpolarized resting membrane potential. Our results suggest that astrocytic TCF7L2 is crucial in coordinating ion and amino acid transport in adulthood, thereby contributing to maintaining extracellular homeostasis and supporting neuronal function. This study identifies TCF7L2 as a key regulator of astrocyte‐mediated neurophysiological support and underscores the importance of its role in astrocyte maturation during postnatal development.

## Graphical abstract

- Astrocytic TCF7L2 impacts extracellular glutamate and glutamine levels.
- Loss of astrocytic TCF7L2 disrupts extracellular potassium clearance.
- TCF7L2‐dependent astrocytic changes reduce neuronal excitability.

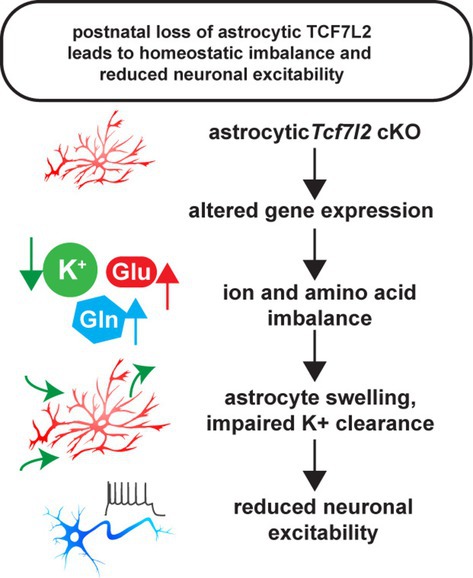

## 1 Introduction

Astrocytes develop throughout the late embryonic and early postnatal development periods (Akdemir et al. 2020), where they actively proliferate and upregulate maturation markers, such as glutamate transporters (Baldwin et al. 2024; Murphy‐Royal et al. 2017), connexins (Mazaud et al. 2021), or potassium channels (Clavreul et al. 2019; Li et al. 2019). Adult astrocytes support neurons in many ways, for example, by regulating their functional maturation through synapse removal (Farizatto and Baldwin 2023; Lee et al. 2021; Vainchtein et al. 2018; Wang et al. 2021) or by providing functional support, for example, reuptake of neurotransmitters (Tan et al. 2021) and potassium clearance (Verkhratsky et al. 2020). Disruptions in astrocyte development during the perinatal period impair their function in adulthood, directly affecting neurons (Kanner et al. 2018; Vivi and Di Benedetto 2024). However, how astrocyte development and maturation are regulated and which internal and external signaling pathways and cellular mechanisms regulate astrocytes are only partially understood.

Astrocyte differentiation and maturation are complex and only partially understood processes, with emerging evidence pointing to the involvement of several transcription factors. Among them, the roles of SOX9 (Cheng et al. 2023; Natarajan et al. 2025), NFIA (Deneen et al. 2006), SOX2 (Wang et al. 2022), and GLI3 (Petrova et al. 2013) have been described. More recently, Wnt/β‐catenin and its effector TCF7L2 have been identified as a key transcription factor in cortical astrocytes (Szewczyk et al. 2023), where it plays a cell‐autonomous role in their postnatal maturation. Loss of astrocytic TCF7L2 has been shown to impair excitatory cortical synapse function and alter social behaviors in adulthood. However, whether these deficits represent the sole consequences of disrupted astrocytic differentiation and maturation due to the absence of TCF7L2 remains unknown.

Astrocytes, through the various channels, transporters, and receptors they express, regulate ion balance and the volume and composition of the extracellular space (ECS) (Simard and Nedergaard 2004). Optimal ECS composition is essential for proper neuronal function and requires continuous regulation. Astrocytes actively control extracellular concentrations of ions and amino acids (AAs) via potassium channels (KCN family), and solute carrier (SLC) transporters (Reed and Blazer‐Yost 2022). For instance, when extracellular K^+^ or glutamate levels rise during neuronal depolarization, astrocytes take them up with water, leading to cell swelling (Risher et al. 2009). This dynamic volume regulation is crucial for maintaining a stable neuronal environment. However, impaired astrocytic function can result in the pathological accumulation of ions or AAs in the ECS, with detrimental consequences that may lead to neuronal dysfunction or damage (Lafrenaye and Simard 2019; Pasantes‐Morales et al. 2000).

This study implicates a novel role for Wnt/β‐catenin signaling and its effector, TCF7L2 transcription factor, in regulating astrocyte function, particularly in maintaining extracellular homeostasis and controlling neuronal excitability. We utilized a previously generated mouse model that allows for the time‐specific deletion of *Tcf7l2* in astrocytes. Our results demonstrate that astrocyte‐intrinsic TCF7L2 is essential for the transcriptional regulation of astrocytic genes involved in AA and ion transport. Dysregulation of these genes led to astrocyte swelling, with consequences including impaired neuronal excitability and a hyperpolarized resting membrane potential in cortical pyramidal neurons. Our findings suggest that the Wnt/β‐catenin signaling effector TCF7L2 may contribute to the regulation of neuronal excitability and is particularly relevant during the postnatal period of astrocyte maturation, potentially influencing long‐term aspects of neuronal physiology.

## 2 Methods

### 2.1 Mice

We used the C57BL/6NTac‐Tcf7l2^tm1a^(EUCOMM)Wtsi/WtsiIeg (*Tcf7l2* ^*tm1a*^) mouse strain (Skarnes et al. 2011) with a trap cassette that was upstream of critical exon 6 of the *Tcf7l2* gene. Mice that were homozygous for the *Tcf7l2* ^*tm1a*^ allele have a total knockout of the gene. To generate the *Aldh1l1*Cre‐ER^T2^:*Tcf7l2* ^*fl/fl*^ strain, in which *Tcf7l2* knockout is induced in astrocytes after the administration of tamoxifen (Merck Life Science, catalog no. T5648‐1G, 75 mg/kg body weight), *Tcf7l2* ^tm1a/+^ animals were first crossed with flippase‐expressing mice (ROSA26:FLPe knock‐in strain; Jackson Laboratory, catalog no. 009086) and then with B6;FVB‐Tg(Aldh1l1‐cre/ERT2)1Khakh/J mice (Jackson Laboratory, catalog no. 029655) that express Cre recombinase from the *Aldh1l1* promoter. *Aldh1l1*WT:*Tcf7l2* ^*fl/fl*^ animals were used as controls. To generate the *Aldh1l1*Cre‐ER^T2^:*Tcf7l2* ^*fl/fl*^:*tdTomato* ^WT/fl^ reporter strain, *Aldh1l1*Cre‐ER^T2^:*Tcf7l2* ^*fl/fl*^ mice were crossed with homozygous Ai9(RCL‐tdT) mice (B6.Cg‐Gt[ROSA]26Sortm9[CAG‐tdTomato]Hze/J; Jackson Laboratory, catalog no. 007909). B6J.129(B6N)‐Gt(ROSA)26Sortm1(CAG‐cas9*‐EGFP)Fezh/J transgenic mice (Jackson Laboratory, catalog no. 026175) encode bicistronic Cas9 and the EGFP cassette, the expression of which is under the control of the CAG promoter is induced by Cre‐mediated STOP cassette removal. For the experimental procedures, all mice were selected by PCR‐based genotyping: *Tcf7l2tm1a* and *Tcf7l2fl* alleles (tcf_F, GGAGAGAGACGGGGTTTGTG; tcf_R, CCCACCTTTGAATGGGAGAC; floxed_PNF, ATCCGGGGGTACCGCGTCGAG; Tm1c_R, CCGCCTACTGCGACTATAGAGA), *Aldh1l1*_Cre allele (31091, CAACAGGTGCCTTCCA; 30308, GGCAAACGGACAGAAGCA), tdTomato (oIMR9105, CTGTTCCTGTACGGCATGG), WPRE (oIMR9103, GGCATTAAAGCAGCGTATC).

In all experiments, both male and female mice were included. The proportion of sexes was balanced across control and cKO groups to avoid sex‐related bias in the analyses. All animals were housed under standard laboratory conditions (21°C ± 2°C, 60% ± 10% humidity, and 12 h/12 h light/dark cycle) with food and water provided *ad libitum*. All experimental procedures were conducted in compliance with current normative standards of the European Community (86/609/EEC) and Polish government (Dz.U. 2015 poz. 266). All protocols for animal use were approved by the Polish Local Ethical Committee No. 1 in Warsaw (permission number 1227/2021). Animal use was controlled by the institutional advisory board for animal welfare at the Centre of New Technologies, University of Warsaw.

### 2.2 Mouse Brain Fixation

Mice at P30 were anesthetized with a mixture of ketamine (Biowet, 125 mg/kg body weight) and xylazine (Biowet, 10 mg/kg body weight). Next, the mice were transcardially perfused with PBS, followed by 4.5% PFA (Merck Life Science, catalog no. P6148) in PBS. Brains were dissected, incubated overnight in 4.5% PFA, and saturated with 30% sucrose (Merck Life Science, catalog no. 1076515000) in PBS at 4°C for 24 h. Next, the brains were transferred into O.C.T. (Sakura Tissue‐Tek, catalog no. 4583) and frozen in −30°C isopentane (VWR, catalog no. 24872.298). Sections were obtained using a Leica CM1860 cryostat. Tissue sections (30 μm) were collected as free‐floating sections in anti‐freeze solution (30% sucrose, Merck Life Science, catalog no. 1076515000; 30% glycerol, VWR, catalog no. 443320113) in 0.1 M PBS (pH 7.4).

### 2.3 Immunohistochemistry

Free‐floating sections were washed three times with PBST. Antigen retrieval was performed using sodium citrate buffer (Bioshop Life Science Products, catalog no. CIT001.1, 10 mM concentration; Tween 20, VWR, catalog no. 663684B, 0.05% concentration). Next, slices were blocked in 10% donkey serum in PBST and incubated overnight with primary antibodies. The next day, the slices were washed and incubated with Alexa Fluor‐conjugated secondary antibodies. Finally, slices were mounted on glass slides with Fluoromount G. Images were captured with an Axio Imager Z2 LSM 700 Zeiss confocal microscope.

### 2.4 Preparation of Transfer Plasmids for rAAV1/2 Production

pZac2.1gfaABC1D‐tdTomato plasmid (TdTomato plasmid) was purchased from Addgene. pAAV:ITR‐U6‐sgRNA(anti‐*lacZ*)‐gfaABC1D‐Cre (control) and pAAV:ITR‐U6‐sgRNA‐(anti‐*Tcf7l2*)‐gfaABC1D‐Cre (*Tcf7l2* KO) transfer plasmids were constructed on a backbone of AAV:ITR‐U6‐sgRNA(backbone)‐hSyn‐Cre‐2A‐EGFP‐KASH‐WPRE‐shortPA‐ITR (Addgene, catalog no. 60231) (Platt et al. 2014), from which 2A‐EGFP‐KASH sequences were removed and the hSyn promoter was replaced by gfaABC1D that was cloned from pZac2.1 gfaABC1D‐tdTomato (Addgene, catalog no. 44332) (Shigetomi et al. 2013). The pAAV:ITR‐U6‐sgRNA‐hSyn‐Cre transfer plasmid had only the 2A‐EGFP‐KASH sequence removed, allowing for guide efficiency tests on thalamic (TCF7L2‐rich) neuronal cells (Szewczyk et al. 2023). To construct the control plasmids, a guide sequence against *lacZ* (TGCGAATACGCCCACGCGAT) was cloned to be expressed in tandem with the gRNA scaffold under control of the hU6 promoter. For the experimental plasmids, the hU6‐sgRNA cassette was duplicated, and a different guide sequence against critical exon 6 of the *Tcf7l2* gene (CGTCAGCTGGTAAGTGCGG; GGTGGGGGTGTTGCACCAC) was cloned into each. Guide sequences were designed with CHOPCHOP v3 Broad Institute GPP sgRNA Designer (https://portals.broadinstitute.org/gpp/public/analysis‐tools/sgrna‐design) and CRISPOR.

### 2.5 Production, Purification, and Titration of rAAV1/2 Particles

HEK293T cells in the exponential growth phase were simultaneously transfected with pAAV1 and pAAV2 rep/cap plasmids, pDF6 helper plasmid, and pAAV:ITR‐U6‐sgRNA‐(anti‐*Tcf7l2*)‐gfaABC1D‐Cre (*Tcf7l2* KO plasmid) for the generation of AAVs *Tcf7l2* KO, pAAV:ITR‐U6‐sgRNA‐(anti‐*lacZ*)‐gfaABC1D‐Cre (Control plasmid) for the generation of AAVs Control, pZac2.1gfaABC1D‐tdTomato (TdT‐plasmid) for the generation of AAVs TdT, and pZac2.1gfaABC1D‐eGFP (eGFP plasmid) for the generation of AAVs GFP using PEI MAX MW 40,000 reagent (Polysciences, catalog no. 24765‐1). pAAV1, pAAV2, and pDF6 plasmids were gifts from Lukasz Swiech (Broad Institute of MIT and Harvard, Cambridge, MA, USA). After 48–72 h, the cells were collected and lysed with sodium deoxycholate (Sigma‐Aldrich, catalog no. D6750, concentration 0.5%). Free nucleic acids were digested with Benzonase Nuclease (Sigma‐Aldrich, catalog no. 70746, concentration: 50 units per ml), and leftover cellular components were discarded after centrifugation. The cell extract was then applied at a steady rate of 1 mL/min on a HiTrap Heparin HP Column (Cytiva, catalog no. 17040601), equilibrated with 150 mM NaCl and 20 mM Tris (pH 8.0) buffer. The column was then washed with 20 mM Tris buffer (pH 8.0) with increasing NaCl content (100, 200, and 300 mM), and viral particles were subsequently eluted using higher salt concentrations (400, 450, and 500 mM). Using Amicon Ultra‐4 Centrifugal Filter NMWL 100 KDa (Millipore, catalog no. UFC810024), AAV particles were concentrated, and the buffer in which they were stored was exchanged for 1× PBS. The viral batch was sterilized by passing it through a Nanosep MF 0.2 μm centrifugal filter (Pall, catalog no. ODPTFE02C34), aliquoted, frozen in liquid nitrogen, and stored at −80°C. Titers of the AAV batch were verified by qPCR. Briefly, the AAV aliquot was treated with DNase (Thermo Fisher Scientific, catalog no. AM2238), denatured at 95°C, and incubated with SmaI restriction enzyme (Thermo Fisher Scientific, catalog no. FD0663) according to the manufacturer's instructions. The numbers of DNA‐containing viral particles were measured using the SYBR Green I Master Kit (Roche) and a LightCycler 480 Instrument II (Roche) in a series of the treated AAV dilutions using primers against the WPRE element. Series of transfer plasmid dilutions at known concentrations were used as references, and each sample was in triplicate. The viral batch was used for the experiments if its concentration was > 1 × 10^9^ DNA‐positive rAAV1/2 particles/μL.

### 2.6 In Vivo AAV1/2 Injections

All stereotaxic injections were performed with a stereotaxic apparatus that was equipped with a microdispensing pump (Neurostar, catalog no. SD46) with a Nanofil syringe (World Precision Instruments, catalog no. 09J, 10 μL volume). For perinatal injections of AAV‐CTR and AAV‐cKO, P6 animals of both sexes were anesthetized by hypothermia, and the head of the animal was fixed with a custom clay mold. Next, 0.5 μL of control AAVs or *Tcf7l2* KO AAVs (8 nL/s) was injected in the right ventricle using the following coordinates: anterior/posterior, 1.5 mm from lambda; medial/lateral, 0.9 mm; dorsal/ventral, −1.5 mm. After the procedures, the pups were placed on a 30°C heating pad and returned to their mother. The animals were sacrificed on P30. For perinatal injections of AAVs TdT, P3 animals were anesthetized by hypothermia and fixed with a custom clay mold. Next, 0.5 μL of AAVs TdT (8 nL/s) was injected in the right ventricle using the following coordinates: anterior/posterior, 1.7 mm from lambda; medial/lateral, 0.9 mm; dorsal/ventral, −1.5 mm.

### 2.7 Nuclei Isolation

Nuclei from the somatosensory cortex of 30‐day old three control and three cKO mice were collected and immediately flash frozen in liquid nitrogen. Next, nuclei were extracted using the Nuclei Isolation Kit: Nuclei EZ Prep (Merck cat. number #NUC101‐1KT). Approximately 15–18 mg of the tissue was thawed, diced with a razor blade, and placed in a dounce homogenizer with 1 mL EZ for 20 strokes loose, and 20 strokes tight. Next, the solution was brought to 5 mL of EZ prep buffer, incubated on ice for 5 min, and centrifuged at 600 rcf for 5 min at 4°C. The supernatant was removed, and the pellet containing nuclei was diluted with 5 mL 0.1% BSA (Merck, cat number 98806‐5G) in DPBS (Thermo Fisher, 14040174) containing RNAse inhibitor (Merck, cat. number. 3335402001, final conc. 0.1 U/uL). The solution was strained using a 40 μm cell strainer (Corning Life Science/Falcon, cat. number 352340) and centrifuged for 5 min at 600 rcf and 4°C. After centrifugation, the supernatant was removed, the pellet was resuspended in 5 mL 0.1% BSA (Merck, 98,806‐5G) in PBS containing RNAse inhibitor, and centrifuged again at 600 rcf for 5 min at 4°C. After this, the supernatant was removed, and the pellet was resuspended in 1.8 mL of Nuclei Suspension Buffer (Fluent, PIPseq T20 3′ Single Cell RNA Kit v4.0), strained using a 10 μm cell strainer (pluriSelect Life Science, cat. number SKU 43‐10010‐40) and centrifuged for 5 min at 600 rcf and 4°C. Next, the supernatant was removed, and the pellet was again resuspended with 0.9 mL of Nuclei Suspension Buffer and centrifuged again at 600 rcf for 5 min at 4°C. Finally, the supernatant was removed, the pellet was diluted with 100 μL of Nuclei Suspension Buffer, and nuclei were counted using a hemocytometer.

### 2.8 Single Nucleus RNA‐Sequencing Using Particle‐Templated Instant Partition Sequencing (PIP‐Seq)

Suspension of single nuclei was partitioned into PIPs following the instructions of the manufacturer. Briefly, 40,000–45,000 nuclei of every specimen were added to a tube containing template beads, pipetted, mixed, and then immediately vortexed according to the protocol of Fluent, PIPseq T20 3′ Single Cell RNA Kit v4.0. After reverse transcription in emulsion, whole transcriptome amplification, and library preparation, sequencing was performed with sequencing by synthesis (Illumina) technology to achieve a depth of 20 k reads per nuclei. After sequencing and quality control we recovered 37,820 nuclei from 3 control and 33,008 nuclei from cKO mice. The bioinformatics analysis of sequenced data was initiated with reads alignment and gene assignment using PIPSeeker. The following steps were performed in the R environment using the Seurat framework. Replicates were integrated with Harmony, doublets were removed with DoubletFinder and clusters were found using Seurat's FindClusters function. MAST was used to find the differences between control and cKO.

### 2.9 GO Analysis

GO analysis was performed using Metascape software (http://metascape.org), with both input and analysis species set to *Mus musculus* . Enrichment was calculated against the entire mouse genome as background. Default parameters of Metascape were applied unless otherwise specified, including: Pathway and Process Enrichment: min overlap: 3; *p* value cutoff: 0.01; min enrichment: 1.5.

### 2.10 Sequencing

Libraries were checked with TapeStation 2200 and HS D1000 reagents and sequenced on the Illumina NovaSeq 6000 system, in pair‐end 2 × 100 mode and standard operating procedures, aiming at 20 kR per cell. Demultiplexing was performed using bcl2fastq v2.20.0.422 (Illumina) software and default settings. After reverse transcription in emulsion, whole transcriptome amplification, and library preparation, sequencing was performed with sequencing by synthesis (Illumina) technology to achieve a depth of 20 k reads per nuclei.

### 2.11 Western Blot

Proteins from somatosensory cortex were extracted using ice‐cold RIPA buffer (Tris, Bioshop Life Science Products, catalog no. TRS001.1, 50 mM concentration, pH 7.5; NaCl, Chempur, catalog no. 794121116, 50 mM concentration; NP‐40, Thermo Scientific, catalog no. 85124, 1% concentration; sodium deoxycholate, Merck Life Science, catalog no. D6750, 0.5% concentration; sodium dodecyl sulfate, Bioshop Life Science Products, catalog no. SDS999.500, 0.1% concentration; ethylenediaminetetraacetic acid [EDTA], VWR, catalog no. E177‐500ML, 1 mM concentration; NaF, Merck Life Science, catalog no. 67414‐1ML‐F, 1 mM concentration; cOmplete, EDTA‐free Protease Inhibitor, Roche, catalog no. 4693132001, 1×; phosphatase inhibitor, Roche, catalog no. 4906845001, 1×), centrifuged, and stored at −80°C. Proteins were separated in sodium dodecyl sulfate polyacrylamide gels (Bio‐Rad, catalog no. 1610183) and transferred to nitrocellulose membranes (Bio‐Rad, catalog no. 1620112). Membranes were blocked with 10% nonfat dry milk in PBST (Tween 20, VWR, catalog no. 663684B, 0.1% concentration) and incubated overnight with primary antibodies (1:500 dilution, anti‐actin, Abcam, catalog no. ab129002, 1:500 HepaCAM, ProteinTech 18177‐1‐AP) at 4°C. After washing, the membranes were incubated with secondary antibodies (anti‐rabbit IgG peroxidase antibody, Sigma Aldrich, catalog no. A0545, 1:10000 dilution; anti‐mouse IgG peroxidase antibody, Sigma Aldrich, catalog no. A9044, 1:10000 dilution) for 2 h at room temperature. Staining was then visualized by chemiluminescence. Images were captured using an ImageQuant LAS 4000 (Cytiva). Densitometric analyses were performed using Quantity One 1‐D software (Bio‐Rad).

### 2.12 Electrophysiology

Brain slices (300 μm thick) from control and cKO mice (four animals per genotype) of both sexes on P21–23 were prepared using an ‘along‐row’ protocol in which the anterior end of the brain was cut along a 45° plane toward the midline. Slices were cut, recovered and recorded at 24°C in regular artificial cerebrospinal fluid (ACSF) composed of: 119 mM NaCl, 2.5 mM KCl, 1.3 mM MgSO4, 2.5 mM CaCl2, 1 mM NaH2PO4, 26.2 mM NaHCO3, 11 mM glucose equilibrated with 95/5% O2/CO2. The somata of layer 5a neurons in the somatosensory cortex and granular neurons in the dentate gyrus of the hippocampus from control and cKO mice were targeted for whole‐cell patch‐clamp recording with borosilicate glass electrodes (resistance 4–8 MΩ). The internal solution was composed of: 125 mM potassium gluconate, 2 mM KCl, 10 mM HEPES, 0.5 mM EGTA, 4 mM MgATP, and 0.3 mM NaGTP (pH 7.25–7.35; 290 mOsm). Patch‐clamp recordings were collected with a Multiclamp 700B (Molecular Devices) amplifier and Digidata 1550A digitizer and pClamp10.6 (Molecular Devices). Recordings were sampled and filtered at 10 kHz. Analysis of action potentials was performed in Clampfit 10.6. Intensity to Voltage (I‐V) plots were constructed from a series of current steps in 20 pA increments from −200 to 1000 pA from a holding potential of −75 mV. A sigmoid curve (hill, 3‐parameter, *f* = a*x ^*b*^/(*c* ^*b*^ + *x* ^*b*^), Sigma Plot) was fitted to I‐V of individual neurons and then parameters were compared between the two groups of mice.

### 2.13 Brain Microdialysis in the Mouse

Animals were anesthetized with butorfanol (0.3 mg/kg, s.c.; Orion Pharma, catalog no. 0520462ad) and isoflurane (Piramal Critical Care, catalog no. IN/EL/0035/19/01) inhalation. Once anesthetized, the scalp was shaved and disinfected with Betadine (EGIS Pharmaceuticals, catalog no. 18509) and 70% ethanol. Mice were then positioned in a stereotaxic frame on a temperature‐controlled heating pad to maintain body temperature. Ophthalmic gel (Vidisic) was applied to prevent corneal drying.

A midline incision (~1.5 cm in length) was made to expose the skull, and the periosteum was removed. A small craniotomy was performed at coordinates determined relative to bregma—AP −1.5, ML +2.8, DV −2.0, dividing by the coefficient between lambda and bregma, providing the corrected coordinates for younger animals. A microdialysis probe (6 kDa, 1 mm membrane length, CMA Microdialysis, catalog no. P000082) was slowly inserted according to the coordinates and perfused with artificial cerebrospinal fluid (aCSF, 2.5 μL/min) of the following composition (in mM): NaCl (130), KCl (5), CaCl_2_ (2.5), MgSO4 (1.3), KH_2_PO_4_ (1.25), NaHCO_3_ (26), and D‐glucose (10), equilibrated with 95/5% O2/CO2. The fraction collected for the first hour was considered the time needed for stabilization of the concentration; then four 10‐min fractions were collected constituting the basal level. After this time, the aCSF was changed to another one with a composition containing an increased level of potassium to 50 mM, with the composition: NaCl (85), KCl (50), CaCl_2_ (2.5), MgSO4 (1.3), KH_2_PO_4_ (1.25), NaHCO_3_ (26), and D‐glucose (10), equilibrated with 95/5% O_2_/CO_2_. After 20 min of pulse, the initial aCSF was returned and after 20 min of stabilization, 10‐min fractions were collected. The fractions were immediately deep‐frozen and the mouse was under inhalation anesthesia of isoflurane in air at a level of 1%–1.5% throughout the experiment. At the end of the procedure, animals were euthanized under deep anesthesia by decapitation.

### 2.14 High‐Performance Liquid Chromatography Determination of Amino Acids

AA concentration in microdialysates was measured using HPLC with fluorescence detection (Thermo Fisher) after derivatization in a timed reaction with *o*‐phthalaldehyde (Merck, catalog no. 1.11452) with mercaptoethanol (Sigma Aldrich, catalog no. M6259), exactly as described earlier (Hamdani et al. 2021). Fifty microliter of samples were injected on to a 5 μm Ultrapure Silica C18 Hl column (250 × 4.6 mm, Thermo Fisher), with a mobile phase of 0.075 M KH_2_PO_4_ solution containing 10% v/v methanol, pH 6.2 (solvent A), and methanol (solvent B). The obtained signals were analyzed using Chromeleon Software (Thermo Scientific, version 6.80 SR13) and the concentration was determined based on the standard curve. Baseline AA concentrations were determined based on the measurement of two baseline fractions collected from each animal and expressed as a mean value.

### 2.15 Mass Spectrometry and Potassium Concentration

Before the analysis, a five‐point calibration curve ranging from 0.01 to 10.0 mg/L was prepared using a certified potassium (K) standard solution. The obtained samples (25 μL) were diluted 100‐fold in a 5% nitric acid (HNO_3_) solution and analyzed for potassium (K) content using an inductively coupled plasma atomic emission spectrometer (ICP‐AES), Thermo Scientific iCAP 7400 Duo, equipped with a TELEDYNE CETAC ASX‐560 autosampler. Potassium concentration was determined based on the emission measured at a wavelength of 766.490 nm.

### 2.16 Quantification of Volume and Area of Astrocytes From AAV‐TdT CTR and AAV‐TdT Animals

Astrocyte morphology, including volume and surface area, was assessed in 30‐μm‐thick floating sections of the mouse somatosensory cortex collected from AAV‐TdT CTR and AAV‐TdT cKO animals at postnatal Day 30 (P30). High‐magnification images were acquired using a confocal microscope (Zeiss Axio Imager Z2 LSM 700) with a 63× objective, scan zoom *X*: 0.7, *Y*: 0.7, and z‐stack of 28–31 slices (27–30 μm in the *z*‐dimension), with an image size of 143 μm × 143 μm. The following criteria were applied for inclusion: the astrocyte cell body was located in the center of the acquired field, and a minimum of 12 μm of astrocyte branches and processes were captured in both directions.

The volume of astrocytes was analyzed using the MeasurementPro package in Imaris 8.4.2 software (Bitplane AG) by creating a three‐dimensional surface rendering of individual astrocytes using whole‐cell surface reconstruction with the surface creation tool (surface detail 0.25 μm). Renders were created based on the pixel gradient intensity algorithm, and TdTomato (595 nm channel, AAV‐TdT CTR and AAV‐TdT cKO) was used to mask individual astrocytes. Renders were thresholded to ensure all processes of astrocytes were properly reconstructed and maintained consistently thereafter.

### 2.17 Quantification of Volume and Area of Astrocytes From AAV‐CTR and AAV‐KO Animals

Astrocyte morphology, including volume and surface area, was assessed in 30‐μm‐thick floating sections of the mouse somatosensory cortex collected from AAV‐CTR and AAV‐KO animals at postnatal day 30 (P30). High‐magnification images were acquired using a confocal microscope (Zeiss Axio Imager Z2 LSM 700) with a 40× objective, scan zoom *X*: 1.4, *Y*: 1.4, and z‐stack of 21–23 slices (20–22 μm in the *z*‐dimension), with an image size of 114 μm × 114 μm. The following criteria were applied for inclusion: the astrocyte cell body was located in the center of the acquired field, and a minimum of 10 μm of astrocyte branches and processes were captured in both directions.

The volume of astrocytes was analyzed using the MeasurementPro package in Imaris 8.4.2 software (Bitplane AG) by creating a three‐dimensional surface rendering of individual astrocytes using whole‐cell surface reconstruction with the surface creation tool (surface detail 0.223 μm). Renders were created based on the pixel gradient intensity algorithm, eGFP (488 nm channel, AAV‐CTR and AAV‐KO) was used to mask individual astrocytes. Renders were thresholded to ensure all processes of astrocytes were properly reconstructed and maintained consistently thereafter.

### 2.18 Astrocyte 3D Morphology—Quantification of Astrocyte Volume and Area Using From CTR and cKO Animals Using a Virtual Reality Environment

Morphology of astrocytes, including volume and surface area, was assessed in 30 μm‐thick floating sections of mouse somatosensory cortex collected from CTR‐TdT and cKO‐TdT at postnatal Day 30 (P30). High magnification images were acquired on a Confocal Microscope—Zeiss Axio Imager Z2 LSM 700, 63× objective, scan zoom *X*: 0.5, *Y*: 0.5, z‐stack 28–29 slices (27–28 μm in the *z* dimension), image size 203 μm × 203 μm. The subsequent analysis was automated using a machine learning algorithm and was therefore not subject to operator blinding. In detail the astrocyte volumes and surface areas were calculated using a machine learning pipeline composed of two independent neural networks, followed by computer vision post‐processing operations. The 3D ground truth annotations were created within a Virtual Reality environment provided via *Oculus Quest*. A dedicated application capable of collecting 3D segmentation annotations was developed for this particular research. To preserve only the most useful data in the context of calculating volumes of astrocytes, we decided to exclude instances located near the edges of a sample. The first part of the pipeline was based on the 3D version of U‐net, provided by the MONAI framework. The U‐net component was responsible for semantic segmentation, that is, distinguishing astrocytes from the background. The neural network's output was used to determine pixel intensity values, that is, the per‐sample threshold between the background and cells. For the complete dataset, the pixel intensity value mean was equal to 8.125, and its standard deviation is equal to 1.166. The second pipeline component identifies the center of mass of each individual cell instance. To achieve that, the astrocyte bodies were enclosed in bounding boxes. The bounding boxes, in turn, were used as training inputs for a 3D version of the RetinaNet model. To reduce the number of false positives in the inference process a high confidence threshold, equal to 0.8, was chosen. The astrocyte bodies were found using the watershed algorithm. Centers of mass, calculated by RetinaNet, were treated as watershed markers, and pixel intensity thresholds, identified by U‐net, as the lowest instance intensity. The watershed part created instance segmentation masks which were then used for calculating astrocyte volumes. The volumes were computed by multiplying the volume of a single voxel by its quantity. Voxel dimensions were collected from sample metadata provided by the microscope. The individual masks were then converted into 3D meshes using the marching cubes algorithm implementation of the scikit‐image library. The meshes were then used to calculate the astrocyte surface areas by summing up all areas of triangles which were building a given 3D mesh. The research data were composed of eight samples generated by the microscope, five samples were used as a train set and the rest of the two samples as a test set. Overall the processing pipeline method, described here, reached 0.891 precision and 0.875 recall for object detection and a 0.671 IoU score for true positives segmentation on the test set. Only true positives were used for final volume and area calculations.

### 2.19 Sholl Analysis of Astrocytes

Astrocyte z‐stack images were thresholded, traced and skeletonized using the SNT plugin in Fiji. Sholl analysis was conducted from the center of the soma at 2 μm intervals, recording the number of intersections. The results were compared separately for each interval and are presented as the mean of five biological replicates (*n* = 5), with 2–3 astrocytes analyzed per replicate.

### 2.20 Statistics, General Methods, and Randomization

GraphPad Prism 9.5.1 software was used for statistical analyses. Data were analyzed using: two‐tailed Mann–Whitney test, or two‐tailed unpaired *t*‐test with Welch's correction. For every figure, statistical tests were justified as appropriate and data met the assumptions of used tests. Individual data points are represented by dots in charts. The statistical tests are described in the figure legends. *p* < 0.05 was considered significant. Outliers were excluded using Grubbs' test. Sample size was chosen based on previous experience and published studies of the authors. No statistical method was used to estimate the size of compared groups Randomization was not necessary.

### 2.21 List of Catalog Numbers and Identifiers

Comprehensive lists of mouse strains, plasmids, antibodies, assays, kits, software, and chemical compounds used in this study, including their sources and identifiers, are provided in Tables S1–S8.

### 2.22 AI‐Assisted Language Editing

The authors used OpenAI's ChatGPT (GPT‐5, April 2025) to assist with English language editing, grammar correction, and stylistic refinement during manuscript preparation. All content was carefully reviewed, verified, and approved by the authors to ensure scientific accuracy and appropriateness. The use of this tool does not affect the authors' accountability for the content of the work.

## 3 Results

### 3.1 Single‐Nucleus RNA‐Sequencing of the Somatosensory Cortex of *Tcf7l2* cKO Mice

**FIGURE 1:**
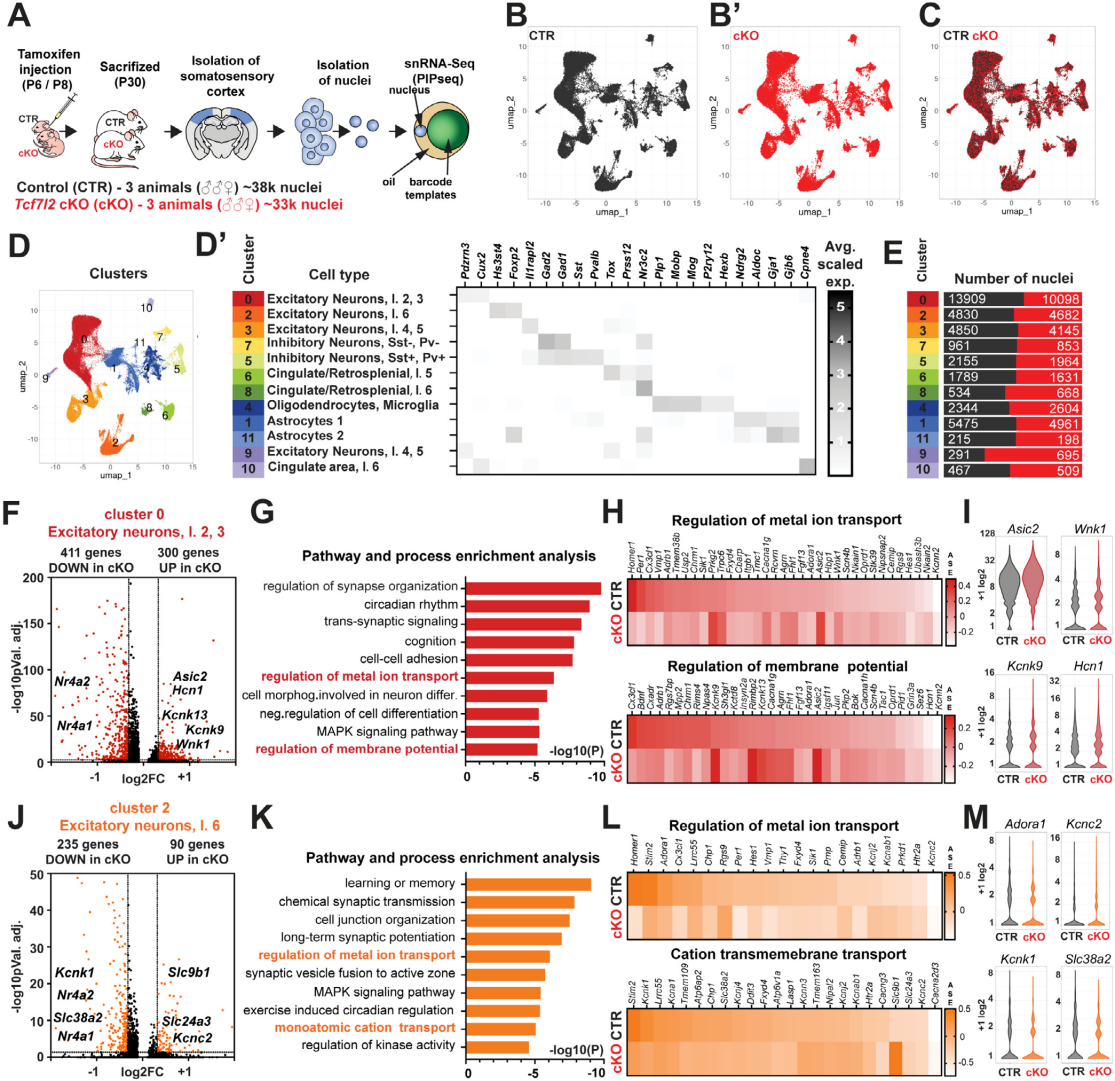
Dysregulation of genes involved in ion exchange in neurons in *Tcf7l2* cKO Mice. (A) A scheme of the experiment of single‐nucleus RNA‐sequencing of somatosensory cortex from control (CTR) and *Tcf7l2* cKO mice using particle‐templated instant partition sequencing (PIP‐seq). (B) UMAP clustering 37,820 nuclei pooling from three CTR mice, P30. (B′) UMAP clustering 33,008 nuclei pooling from three cKO mice, P30. (C) UMAP clustering CTR and cKO nuclei (black—CTR, red—cKO) (D) Clusters of CTR and cKO nuclei colored by cell type (D′) corresponding average scaled expression (ASE) heatmap enriched/unique transcripts per each identified cluster. (E) Quantification of cluster composition by a number of identified nuclei (black—CTR, red—cKO). (F) Volcano plot of the differentially expressed genes in CTR and cKO cortical neurons, layers 2 and 3 (red: *p* adj. < 0.05, 0.8 > Fold Change > 1.25). (G) 10 out of 20 top Gene Ontology (GO) terms for down‐ and upregulated genes in cKO cortical neurons, layers 2 and 3. (H) GO‐associated differentially expressed genes presented as average scaled expression (ASE) heatmaps (CTR upper, cKO lower). (I) Violin plots of picked differentially expressed genes between CTR and cKO astrocyte clusters. (J) Volcano plot of the differentially expressed genes in CTR and cKO cortical neurons, layer 6 (orange: *p* adj. < 0.05, 0.8 > Fold Change > 1.25). (K) 10 out of 20 top Gene Ontology (GO) terms for down‐ and up‐regulated genes in cKO cortical neurons, layer 6. (L) GO‐associated differentially expressed genes presented as average scaled expression (ASE) heatmaps (CTR upper, cKO lower). (M) Violin plots of picked differentially expressed genes between CTR and cKO astrocyte clusters.

Astrocytes play a crucial role in shaping synaptic connectivity, and our recent findings uncovered that astrocytic TCF7L2 is involved in cortical synapse development and function (Szewczyk et al. 2023). To uncover previously unidentified mechanisms and processes in the brain involving astrocytic TCF7L2, we performed single‐nucleus RNA sequencing (snRNA‐Seq) of the somatosensory cortex from control and cKO mice at postnatal day 30 (P30) (Figure 1A, Figure S1A,B). To do this, we used a breeding strategy that generated the *Tcf7l2* cKO (cKO—*Aldh1l1*Cre‐ER^T2^:*Tcf7l2* ^*fl/fl*^) genotype and control mice (control—*Aldh1l1*WT:*Tcf7l2* ^*fl/fl*^), with tamoxifen (75 mg/kg body weight) administered on postnatal day six (P6) and eight (P8). Isolated nuclei from the somatosensory cortex of control and cKO mice were subjected to single‐nucleus RNA Particle‐templated instant partition sequencing (PIP‐seq). After sequencing and quality control, we recovered 37,820 control and 33,008 cKO nuclei. The bioinformatics analysis of sequenced data was initiated using PIPSeeker and followed in the R environment using the Seurat framework. Replicates were integrated with Harmony; clusters were found using Seurat's FindClusters function, while MAST was used to find the differences between control and cKO. We identified 12 clusters of nuclei from control and cKO samples using the Seurat software for automatic clustering (Figure 1B,B′,C). We did not identify any clusters unique to cKO mice.

The classification of these clusters into distinct cell types was manually performed based on the expression of well‐established marker genes (Figure 1D,D′, Figures [Link], [Link]). Cluster 0 was identified as layer 2/3 cortical excitatory neurons due to the high expression of *Cux2*, *Pdzrn3*, and *Lamp5*. Cluster 2 was classified as layer 6 cortical excitatory neurons based on the elevated expression of *Foxp2*, *Rell3*, and *Hs3st4*, while cluster number 3 was characterized as layer 4/5 excitatory neurons based on the enriched expression of *Il1rapl2* and *Hs3st2*. Nuclei within clusters 5 and 7 were identified as inhibitory cortical neurons, characterized by high expression of *Gad1* and *Gad2*. Among them, only Cluster 7 exhibited strong expression of *Pvalb* and *Sst*. Cluster 4 consisted of oligodendrocytes and microglia, as indicated by the expression of oligodendrocyte and myelin‐associated genes (*Plp1*, *Mobp*, and *Pdgfra*) and microglial markers (*P2ry12* and *Hexb*.). The majority of astrocytes were found in Cluster 1 (96.1%), characterized by high expression of *Aldoc* and *Ndrg2*, and in Cluster 11 (3.9%), which was enriched in the expression of *Gja1* and *Gjb6*. We did not observe any significant changes in the number of nuclei within cKO clusters compared to controls (Figure 1E). Collectively, those findings show that the absence of *Tcf7l2* in postnatal astrocytes does not lead to the generation of unique or cKO‐specific cell populations in the somatosensory cortex.

### 3.2 Dysregulation of Genes Involved in Ion Exchange in Neurons in *Tcf7l2* cKO Mice

To investigate the consequences of impaired astrocytic maturation caused by lack of postnatal TCF7L2 on cortical neurons, we conducted a detailed analysis of gene expression alterations within identified neuronal clusters using the obtained snRNA‐Seq data. The conditional deletion of *Tcf7l2* in astrocytes led to changes in the expression of 659 genes in neuronal Cluster 0—excitatory neurons, layers 2 and 3; among them, 411 were downregulated and 300 were upregulated in cKO (Figure 1F, Supporting Information, *p* value adjusted 0.05, 0.8 > Fold Change > 1.25). Gene ontology analysis showed the significant enrichment of networks involved in regulating synapse organization and behavior–cognition (Figure 1G, Figure S7A). These findings were expected, as we had previously demonstrated that *Tcf7l2* cKO mice exhibited increased cortical excitatory and inhibitory synapses, as well as marked changes in social interaction by adulthood. However, among 20 of the most significantly enriched GO terms, we also observed networks not previously associated with astrocytic TCF7L2. They were: the regulation of metal ion transport and the membrane potential (Figure 1G). Within these groups, we observed changes in genes that encode potassium channels *Kcnk9*, *Kcnn2*, *Kcnk13*, *Kcnn2*, *Hcn1*, acid‐sensing ion channels—*Asic2*, as well as genes regulating sodium, potassium, and chloride ion transport pathways, for example, serine/threonine kinase *Wnk1* (Figure 1H,I). Beyond ion transport and synaptic regulation, we also detected alterations in signaling pathways, including MAPK, and a downregulation of genes associated with cell–cell communication, such as *Nr4a1*, *Nr4a2*, and *Nr4a3* encoding orphan nuclear receptors. These findings suggest that, beyond its established role in synapse organization and behavior, astrocytic TCF7L2 may contribute to ion homeostasis, mainly by ion transport pathways in neurons directly or indirectly.

Next, to see whether astrocytic TCF7L2 impacts cortical neurons in a layer‐specific manner, we analyzed gene expression in two other neuronal clusters: Cluster 2—excitatory neurons, layer 6, and Cluster 3—excitatory neurons 4 and 5. The conditional deletion of *Tcf7l2* in astrocytes resulted in dysregulated expression of 325 genes in Cluster 2, among which 235 were downregulated and 90 were upregulated (Figure 1J, Supporting Information, *p* value adjusted 0.05, 0.8 > Fold Change > 1.25). As expected, gene ontology analysis showed the significant enrichment of networks involved in synaptic transmission and behavior–learning (Figure 1K, Figure S7B). Similar to the changes observed within Cluster 0, the regulation of metal ion transport and monoatomic cation transmembrane transport were among the enriched processes (Figure 1K). Among dysregulated genes were those regulating potassium ion homeostasis and transport—potassium channels, *Kcnab1*, *Kcnj2*, *Kcnc2 Kcna1*, *Kcnj4*, *Kcnk1*, *Kcnn3*; genes encoding proteins involved in sodium, potassium, calcium, and proton transport, *Fxyd4*, *Chp1*, *Adora1* and AA transport *Slc38a2*, *Slc9b1*, *Slc24a3* or those involved in modulation of membrane potential, for example, *Adrb1* (Figure 1L,M). Like in Cluster 0, genes involved in MAPK signaling and encoding orphan nuclear receptors were dysregulated.

Analysis of Cluster 3 (cortical excitatory neurons, layer 4 and 5) showed dysregulation of the expression of 455 genes; 364 were downregulated, while 91 were upregulated in cKO neurons (Figure S7C, Supporting Information, *p* value adjusted 0.05, 0.86 > Fold Change > 1.15). Similarly to changes in Cluster 0 and 2, gene ontology analysis showed the significant enrichment of networks such as synaptic signaling, behavior–cognition (Figure S7D), as well as regulation of synaptic membrane potential, cation transport *Atp1a3*, potassium channels *Kcnmb4*, *Kcnk2*, *Kcnk9*, *Kcnj3*, *Kcnn2*, *Hcn1*, regulation of AA transport, for example, *Slc38a2* (Figure S7E–G).

These findings suggest that astrocytic TCF7L2 plays a crucial role in neuronal synaptic regulation and ion transport, independent of cortical layer specificity. The observed widespread alterations in the expression of genes encoding potassium and sodium channels or AA transporters suggest a potential link through which astrocytic TCF7L2 dysfunction may contribute to neuronal impairments.

### 3.3 *Tcf7l2* Regulates Astrocytic Genes Involved in Cell Homeostasis and Brain Osmoregulation

**FIGURE 2:**
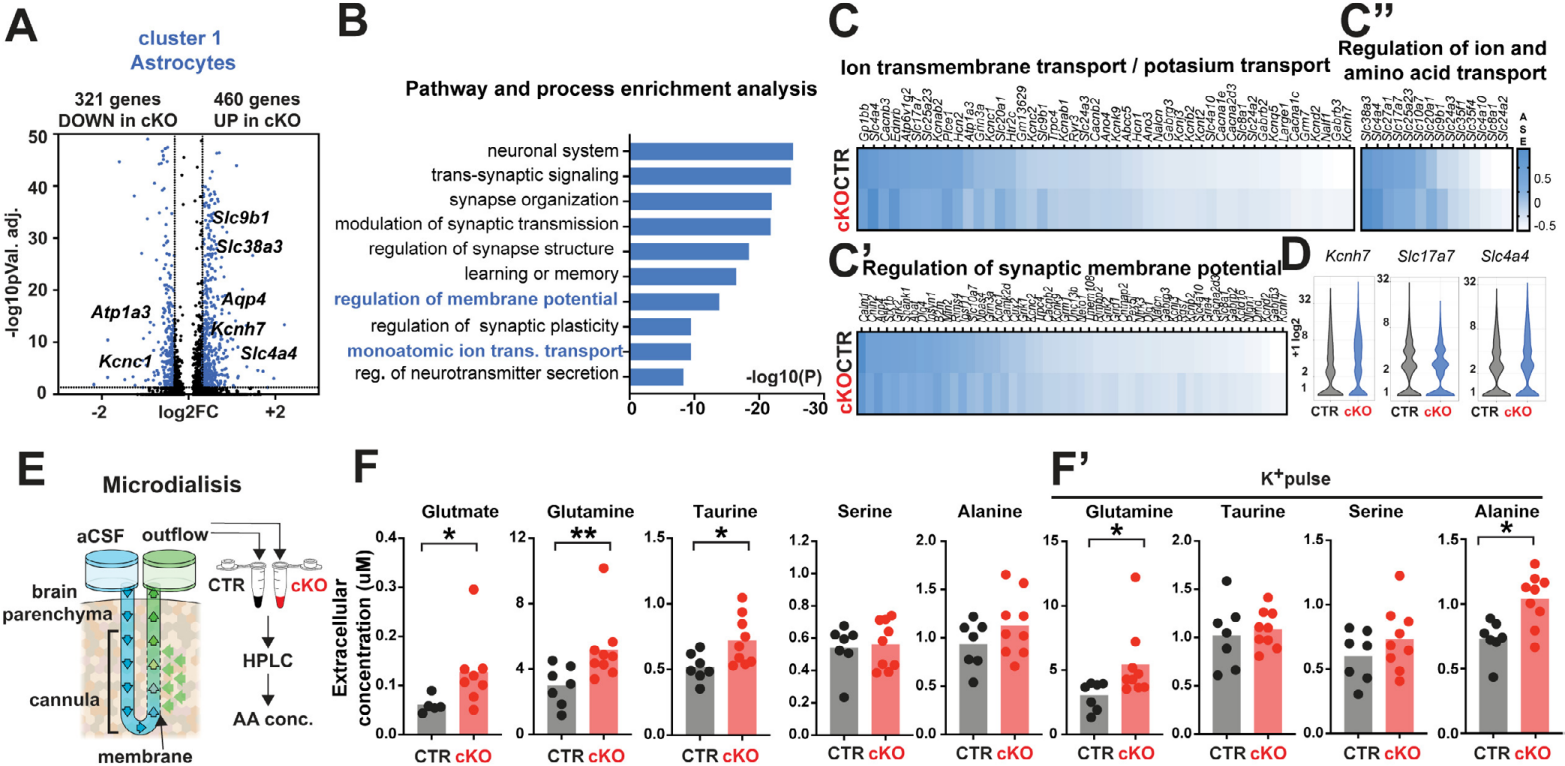
Dysregulation of the amino acids homeostasis in the somatosensory cortex of *Tcf7l2 cKO Mice*. (A) Volcano plot of the differentially expressed genes in control and cKO astrocytes (blue: *p* adj. < 0.05, 0.8 > Fold Change > 1.25). (B) 10 out of 20 top Gene Ontology (GO) terms for down‐ and up‐regulated genes in the cKO astrocytic cluster. (C, C″) GO‐associated differentially expressed genes presented as average scaled expression (ASE) heatmaps (CTR upper, cKO lower). (D) Violin plots of picked differentially expressed genes between CTR and cKO astrocyte clusters. (E) A scheme of the microdialysis in the cortex of CTR and cKO mice, aCSF – artificial cerebrospinal fluid, extracellular amino acids level in microdialysates was measured in the somatosensory cortex of CTR and cKO mice before and 30 min after potassium pulse (K+) using high‐performance liquid chromatography. (F) Concentration of glutamate, glutamine, taurine, serine and alanine in microdialysis of CTR and cKO mice. Dots represent individual mice. The data are expressed as the mean. The data were analyzed using a two‐tailed Mann–Whitney Test. (F′) The concentration of glutamate, glutamine, taurine, serine, and alanine in microdialysis of CTR and cKO mice 40 min after a potassium pulse (K+). Dots represent individual mice. The data are expressed as the mean. The data were analyzed using a two‐tailed Mann–Whitney Test.

Given the significant transcriptional changes observed in neurons, we assumed these were driven by upstream alterations in astrocytic function. Next, we examined gene expression differences between control and cKO astrocytic populations to check this. The conditional deletion of *Tcf7l2* led to changes in the expression of 781 genes in astrocytic Cluster 11, among which 321 were downregulated and 460 were upregulated in cKO nuclei compared to control (Figure 2A, Supporting Information, *p* value adjusted 0.05, 0.8 > Fold Change > 1.25.). As expected, differential gene expression and Gene Ontology (GO) analysis revealed a robust dysregulation of genes related to synaptic processes, for example, trans‐synaptic signaling, synapse organization, modulation of chemical synaptic transmission, and regulation of synapse structure or activity (Figure 2B). However, among dysregulated genes in the cKO astrocytic cluster, we observed a high number of genes involved in the modulation of membrane potential and in the regulation of monoatomic ion transmembrane transport/monoatomic cation transport. Among the latter they were, for example, sodium transporters: *Slc9b1*, *Atp1a3*, *Slc8a1*, *Nalcn*; calcium transporters: *Slc24a2*, *Trpc4*, *Cacna1e*, *Cacnb2*, *Cacna1c*, *Cacna2d3*, *Cacnb3*, *Ryr3*, *Slc24a3*; H+ transporters: *Slc4a10*, *Slc4a4*, *Atp6v1g2*; and potassium transporters: *Kcnt2*, *Kcnh7*, *Kcnk9*, *Kcnb2*, *Kcnc1*, *Kcnd2*, *Kcnj3*, *Kcnab1*, *Kcnab2*, *Kcnq5*, *Kcnc2* (Figure 2C,C′). One striking observation was the high number of dysregulated genes encoding SLC superfamily transporters, which have emerged as crucial players in maintaining cellular ion and AA homeostasis (*Slc10a7*, *Slc25a23*, *Slc27a1*, *Slc17a7*, *Slc24a2*, *Slc4a4*, *Slc24a3*, *Slc38a3*, *Slc4a10*, *Slc35f1*, *Slc35f4*, *Slc8a1*, *Slc20a1*, *Slco3a1*, *Slco1b2*, *Slc9b1*, Figure 2C″,D). Another was the upregulation in the cKO astrocytes *Aqp4* gene, which encodes water channels specifically localized to astrocytes.

Our findings demonstrate dysregulation in genes encoding AAs and ion transporters. This suggests that, impaired astrocytic development due to TCF7L2 deficiency may impact extracellular AA homeostasis and composition.

### 3.4 Dysregulation of the Amino Acid Homeostasis in the Somatosensory Cortex of *Tcf7l2* cKO Mice

To test whether the ECS composition and homeostasis are affected in *Tcf7l2* cKO mice, the extracellular concentration of selected AAs was measured. For this purpose, we stereotactically implanted into the somatosensory cortex layer 4/5 of control and *Tcf7l2* cKO mice (AP −1.5, ML +2.8, DV −2 with correction of lambda/bregma coefficient) a microdialysis probe equipped with semipermeable dialysis (Figure 2E). We collected samples of cortical extracellular fluid containing, among others, AAs that crossed the dialysis membrane and analyzed their concentration using high‐performance liquid chromatography (HPLC). The HPLC analysis of microdialysates showed an increase in the extracellular concentration of glutamate by ~111% (from 0.060 ± 0.017 mM in control to 0.127 ± 0.033 mM in *Tcf7l2* cKO), glutamine by ~73% (from 2.99 ± 1.21 mM in control to 5.16 ± 1,99 mM in *Tcf7l2* cKO) and taurine by ~39% (from 0.52 ± 0.11 mM in control to 0.72 ± 0.19 mM in *Tcf7l2*) (Figure 2F). We did not observe changes in serine and alanine extracellular concentrations. While basal microdialysis can detect neurotransmitter levels in the ECS, high potassium (50 mM) artificial cerebrospinal fluid (ACSF) perfusion through the probe for 20 min has been used to examine the K^+^—induced release and distribution of metabolites from the somatosensory cortex. The effects of membrane depolarization were measured 40 min after the potassium pulse. After this period, during which the metabolite levels are expected to equilibrate and achieve proper distribution, an increase in the glutamine concentration by ~79% (from 3.03 ± 1.09 mM in control to 5.41 ± 2.78 mM in *Tcf7l2* cKO), and alanine by ~42% (from 0.73 ± 0.15 mM in control to 1.04 ± 0.20 mM in *Tcf7l2* cKO), was observed. We did not observe taurine and serine extracellular concentration changes after potassium‐induced membrane (Figure 2F′).

Our findings demonstrate that the postnatal loss of astrocytic TCF7L2 alters the expression of genes encoding AA transporters. These alterations may underlie the observed changes in extracellular AA levels, their homeostatic regulation, and spatial distribution. These findings raise a question: What are the cellular and physiological consequences of such dysregulation in the cortex of *Tcf7l2* cKO mice?

### 3.5 Astrocyte Swelling in *Tcf7l2* cKO Mice

**FIGURE 3:**
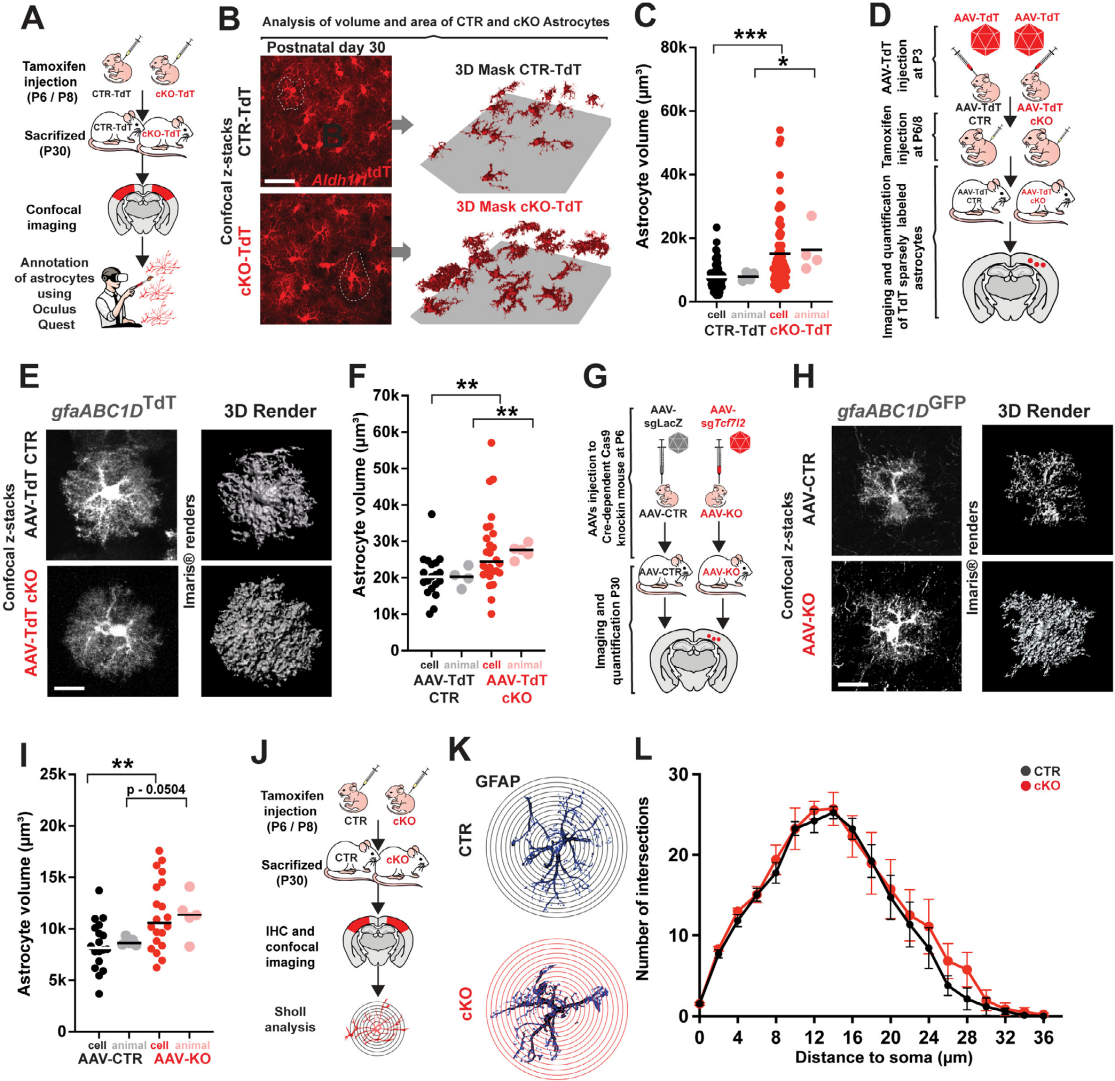
Astrocyte swelling in the cortex of *Tcf7l2* cKO Mice. (A) Schematic of the experiment of analysis of volume and area of TdT‐CTR and TdT‐cKO astrocytes using virtual reality via Oculus Quest and machine learning algorithm. (B) Representative images of TdTomato+ astrocytes in the somatosensory cortex of TdT‐CTR and TdT‐cKO mice showing raw images (left) and 3D rendering (right); Scale = 20 μm. (C) Quantification of the volume of TdTomato+ astrocytes in the somatosensory cortex of TdT‐CTR and TdT‐cKO mice. Black and red dots represent individual astrocytes, while gray and pink represent individual animals; *n* = 62 astrocytes from 4 mice (TdT‐CTR), *n* = 74 astrocytes from 4 mice (TdT‐cKO). The data were analyzed using the two‐tailed Mann–Whitney Test. (D) Schematic of the in vivo microinjection of AAVs that expressed TdTomato fluorescent protein under the astrocyte‐specific *gfaABC1D* promoter on P3 in CTR versus cKO pups, followed by a tamoxifen injection on P6 and P8. (E) Representative images of TdT+ astrocytes in the somatosensory cortex of AAV‐TdT CTR and AAV‐TdT cKO mice (left) and their three‐dimensional Imaris renders (right). Scale bar = 20 μm. (F) Quantification of the volume of TdTomato+ astrocytes in the somatosensory cortex of AAV‐TdT CTR and AAV‐TdT cKO mice. Black and red dots represent individual astrocytes, while gray and pink represent individual animals. Calculation of the volume of individual astrocytes was performed in Imaris based on three‐dimensional renders of *n* = 19 astrocytes from 4 AAV‐TdT CTR mice, *n* = 25 astrocytes from 5 AAV‐TdT cKO mice. An unpaired *t*‐test was performed to analyze the differences between groups. (G) Schematic of the in vivo adeno‐associated (AAV)‐mediated deletion of *Tcf7l2* in postnatal astrocytes. (H) Representative confocal images of GFP+ astrocytes in the somatosensory cortex of AAV‐CTR and AAV‐KO mice (left) and their three‐dimensional Imaris renders (right). Scale bar = 10 μm. (I) Quantification of the volume of GFP+ astrocytes in the somatosensory cortex of AAV‐CTR and AAV‐KO. Black and red dots represent individual astrocytes, while gray and pink represent individual animals. Calculation of the volume of individual astrocytes was performed in Imaris based on three‐dimensional renders of *n* = 18 astrocytes from 5 AAV CTR mice, *n* = 21 astrocytes from 5 AAV KO mice. The data were analyzed using a two‐tailed Mann–Whitney Test. (J) Schematic of the experiment of Sholl analysis of TdT + CTR and TdT + cKO astrocytes using the SNT plugin in Fiji. (K) Binary (thresholded) representations of astrocytes after skeletonization with SNT in the somatosensory cortex of CTR or *Tcf7l2* cKO (below). (L) Sholl plot of CTR and cKO astrocytes, *n* = 5. The data were analyzed using a two‐tailed Mann–Whitney Test.

The AA distribution in the brain tissues and subcellular fractions impacts cell volume. Since AAs are osmotically active, they may affect water movement across the cell membrane, leading to cell swelling and, consequently, cell edema (Pasantes‐Morales et al. 2000). To assess the potential influence of dysregulated AA homeostasis on cortical astrocyte morphology, we examined cell volume and area in control and cKO mice. To do this, we used astrocyte‐specific *Tcf7l2* conditional knockout with incorporated a cytoplasmic astrocyte TdTomato reporter, cKO‐TdT (*Aldh1l1*Cre‐ER^T2^:*Tcf7l2* ^*fl/fl*^:*TdTomato* ^*WT/fl*^), and a control line—CTR‐TdT—(*Aldh1l1*Cre‐ER^T2^:*Tcf7l2* ^*WT/WT*^:*TdTomato* ^*WT/fl*^). Thirty days old CTR‐TdT and cKO‐TdT were sacrificed; brains were collected and visualized using confocal microscopy (Figure 3A). Next, we developed a method based on a machine learning algorithm that allows us first to distinguish an individual *Aldh1l1* ^TdT+^ astrocyte within collected z‐stacks of somatosensory cortex of cKO‐TdT and CTR‐TdT mice (Figure 3B) and generate a 3D mask to quantify their volume and cell surface area. Quantification showed that the volume (Figure 3C) and cell area (Figure S8A) of cKO astrocytes are increased compared to control cells. We also analyzed the cell soma volume separately, which showed soma swelling in cKO astrocytes (Figure S8B).

Next, to independently validate these findings and further establish the robustness of our approach, we used a classic method of quantifying cell volume and area based on three‐dimensional renders of TdT+ astrocytes using Imaris Software. In this experiment, we used a breeding strategy that let us generate the *Tcf7l2* cKO (*Aldh1l1*Cre‐ER^T2^:*Tcf7l2* ^*fl/fl*^) genotype and control mice (*Aldh1l1*WT:*Tcf7l2* ^*fl/fl*^) without robustly incorporating a fluorescent dye. To sparsely label cortical astrocytes with TdTomato fluorescent protein, on Day 3, we intraventricularly injected AAVs‐TdT with TdT sequence under the gfaABC1D promoter (Figure 3D), which was followed by tamoxifen administration on P6 and P8. Twenty‐two days later, AAV‐TdT cKO and AAV‐TdT CTR mice were sacrificed, and cortical sparsely labeled astrocytes were visualized under a confocal microscope. Next, we quantified astrocyte volume and area and found that cortical astrocytes from AAV‐TdT cKO mice had significantly larger volumes (Figure 3E,F) and cell areas than controls (Figure S8C). This finding confirmed the validity of our generated model based on machine learning and proved that the lack of TCF7L2 in postnatal astrocytes leads to increased cell volume. Finally, we used an independent genetic model of *Tcf7l2* deficiency in astrocytes, where AAV‐mediated delivery of CRISPR/Cas9 delete *Tcf7l2* effectively in a subset of cortical astrocytes (Figure 3G). Quantification of GFP+ cortical astrocytes from 30‐day‐old AAV‐KO and AAV‐CTR animals, using confocal microscopy and Imaris software, shows significantly larger volume (Figure 3H,I) and area (Figure S8D) in AAV‐KO than in control mice.

Next, to assess astrocytic branching complexity, we performed Sholl analysis based on z‐stack images acquired from the somatosensory cortex, layer 5 (Figure 3J). Using a 2 μm interval from the center of the cell, the analysis revealed no significant morphological differences in astrocytic arborization between the control and cKO groups expressing GFAP protein (Figure 3K,L). When considered alongside data estimating overall astrocyte size, these findings suggest that the observed increase in cell volume is likely due to cellular swelling, rather than changes in branching complexity.

Astrocytes mature morphologically during postnatal cortical development, becoming larger during the first 30 postnatal days (Morel et al. 2017; Stogsdill et al. 2017). One of the mechanisms regulating astrocyte size is the presence of the membrane HepaCAM protein that mediates interactions between neighboring astrocytes. Changes in the HepaCAM level might fail to allow astrocytes to tile correctly, leading to an increase in cell volume (Baldwin et al. 2021). To exclude that possibility, we quantified the astrocytic HepaCAM expression using RT‐PCR and Western blot (Figure S9). We observed no changes in overall mRNA level nor protein in cKO brains, which suggested that the observed astrocytic phenotypes in mice lacking *Tcf7l2* are not caused by HepaCAM‐dependent processes.

Our findings indicate that TCF7L2‐deficient astrocytes may undergo swelling in response to elevated levels of AAs in the ECS. This astrocytic swelling likely reflects a compensatory mechanism to restore brain homeostasis by reducing extracellular AA concentrations and re‐establishing osmotic balance, which has been disrupted due to dysregulation of ion and AA transporter gene expression.

### 3.6 Dysregulation of Potassium Concentration in the Somatosensory Cortex of *Tcf7l2* cKO Mice

**FIGURE 4:**
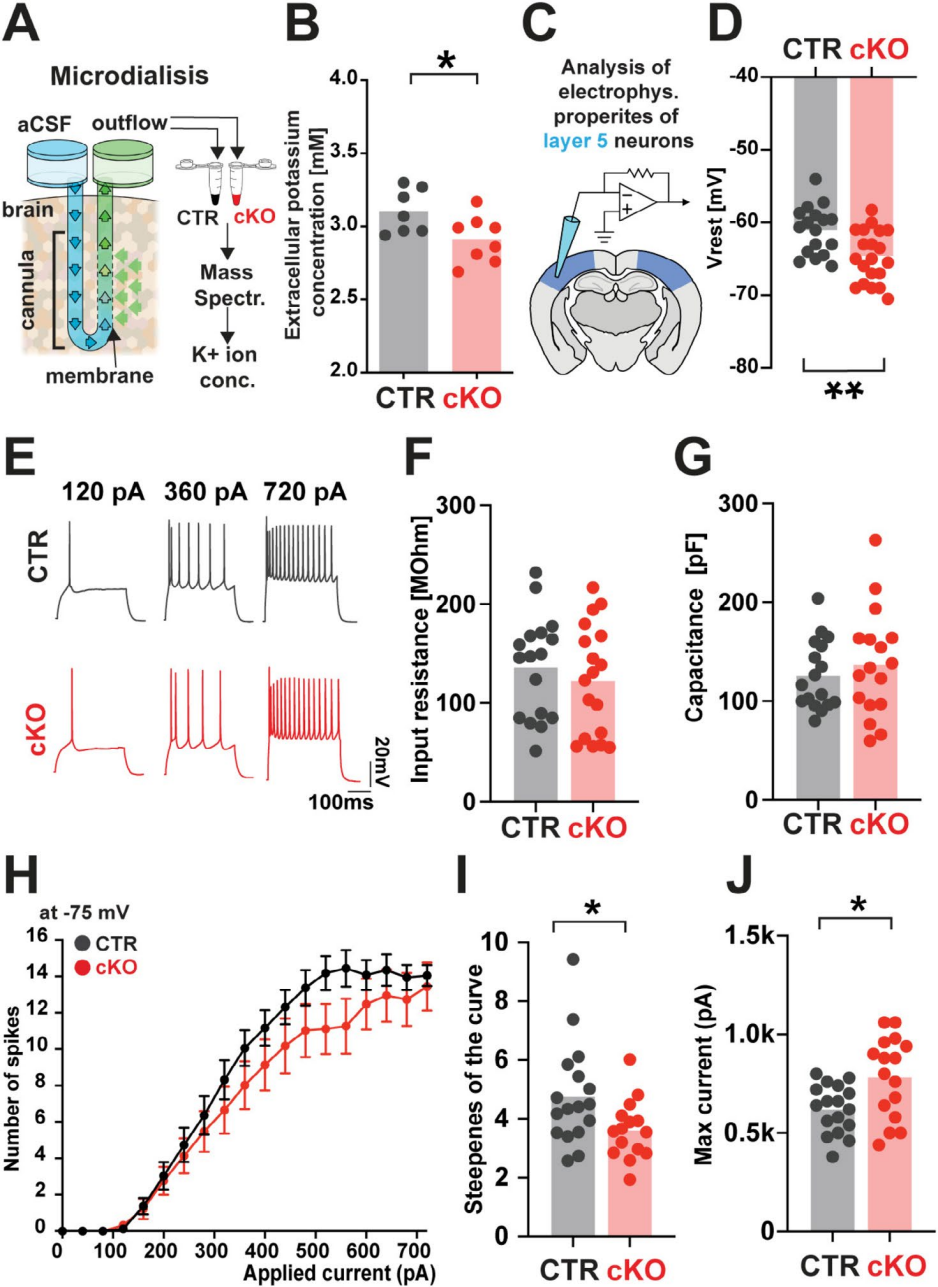
Decreased potassium level in the extracellular space and hyperpolarized cortical neurons in *Tcf7l2* cKO mice. (A) A scheme of the microdialysis in the cortex of CTR and cKO mice, aCSF—artificial cerebrospinal fluid, extracellular potassium level in microdialysates was measured in a somatosensory cortex of CTR and *Tcf7l2* cKO mice 40 min after potassium pulse (K+) using mass spectrometry analysis of potassium ions. (B) The concentration of potassium ions in CTR and cKO microdialysates. Dots represent individual mice. The data are expressed as the mean. An unpaired *t*‐test was performed to analyze group differences after assessing data distribution with the Shapiro–Wilk normality test and lognormality testing. (C) A scheme of whole‐cell patch‐clamp recordings of barrel cortex neurons, layer 5 from CTR and cKO mice. (D) Resting membrane potential in CTR and cKO mice. The data are expressed as the mean number of neurons: *n* = 17 neurons from 4 CTR mice; *n* = 16 neurons from 4 cKO mice. An unpaired *t*‐test was performed to analyze group differences after assessing data distribution with the Shapiro–Wilk normality test and lognormality testing. (E) Representative action potential traces in response to indicated amounts of current in current‐clamp mode from CTR (upper, black) and cKO (lower, red) neurons. (F) Input Resistance mV in CTR and cKO mice. The data are expressed as the mean; number of neurons: *n* = 17 neurons from 4 CTR mice; *n* = 16 neurons from 4 cKO mice. (G) Membrane Capacitance in CTR and cKO mice. The data are expressed as the mean number of neurons: *n* = 17 neurons from 4 CTR mice; *n* = 16 neurons from 4 cKO mice. (H) A number of spikes evoked by increasing depolarizing currents at −75 mV in CTR and cKO neurons. (I) Steepness of the curve in CTR and cKO neurons. An unpaired *t*‐test was performed to analyze group differences after assessing data distribution with the Shapiro–Wilk normality test and lognormality testing. (J) Current at maximum frequency; a unpaired *t*‐test was performed to analyze group differences after assessing data distribution with the Shapiro–Wilk normality test and lognormality testing.

Increased levels of taurine, glutamate, and glutamine—key osmolarity regulators—accompanied by astrocyte swelling and abundant dysregulation of genes encoding potassium channels, raise questions about their collective consequences on the physiology of the cortex and impact on cortical neurons. First, to check if changes in astrocyte volume caused by a lack of *Tcf7l2* can cause changes in extracellular potassium levels, we analyzed the concentration of K+ in the ECS. We stereotactically implanted a microdialysis probe into the somatosensory cortex layer 4/5 of control and *Tcf7l2* cKO mice (AP −1.5, ML +2.8, DV −2 with correction of lambda/bregma coefficient). We applied a pulse of aCSF enriched to 50 mM potassium concentration for 20 min, causing depolarization of biological membranes, and measured potassium levels 20 min after the pulse, a time sufficient for astrocytes to regulate and clear the ECS of excess metabolites and ions (Figure 4A). We observed lower potassium levels in the ECS of the somatosensory cortex in *Tcf7l2* cKO mice when compared to the control (Figure 4B). The results showed that regulation of extracellular potassium levels is impaired in *Tcf7l2* cKO mice. This finding suggests that astrocytic TCF7L2 may contribute to ion homeostasis by changing the level of extracellular potassium.

### 3.7 Astrocytic *Tcf7l2* Restricts Neuronal Excitability

Finally, to test if changes in AAs and potassium levels as well as in genes encoding potassium and sodium channels, could potentially translate into functional changes in neuronal physiology, we performed whole‐cell patch‐clamp recordings in control and *Tcf7l2* cKO mice, focusing on excitatory layer 5 pyramidal neurons in the somatosensory cortex (Figure 4C). We found that neurons from *Tcf7l2* cKO mice had a hyperpolarized resting membrane potential relative to controls (Figure 4D,E), however, no changes in membrane resistance and cell capacitance were noticed (Figure 4F,G), Consistent with changes in resting potential, neurons from *Tcf7l2* cKO were less excitable in response to stimulation (Figure 4H), and required a higher current intensity to reach maximal firing frequency (Figure 4I,J). However, the maximum firing frequencies once achieved were similar in neurons from *Tcf7l2* cKO mice compared to those in controls. Finally, to test if observed changes are specific to cortical neurons, we performed whole‐cell patch‐clamp recordings in control and *Tcf7l2* cKO mice, focusing on hippocampal granular neurons (Figure S10A). No changes in membrane potential and no differences in response to stimulation were observed in hippocampal neurons of cKO mice (Figure S10B–D), suggesting that the observed impairment is cortical‐specific.

Our findings suggest that the postnatal role of astrocytic TCF7L2, beyond its established impact on synapse organization and behavior, may contribute to AA and ion homeostasis, changes in which appear to correlate with impaired neuronal excitability in the somatosensory cortex.

## 4 Discussion

Our study suggests that the astrocytic Wnt/β‐catenin effector TCF7L2 plays an important role in maintaining extracellular brain homeostasis and influencing neuronal excitability. Using a temporally controlled, conditional deletion of the *Tcf7l2* gene, we investigated its role in early postnatal cortical development. We then analyzed young adult mice to assess the consequences of astrocytic TCF7L2 deficiency on cortical neurons and brain function. Our findings reveal a previously unrecognized role for TCF7L2 in astrocyte function and demonstrate that this transcription factor is specifically required during astrocyte development to preserve brain homeostasis and neuronal function.

The specification of embryonic astrocytes (Bayraktar et al. 2014), followed by postnatal differentiation, proliferation (Clavreul et al. 2019), and final maturation, is tightly regulated by the transcription factors (Markey et al. 2023). For instance, NFIA and SOX9 transcription factors interact to mediate the initiation of gliogenesis (Kang et al. 2012), while YAP and TAZ impact progenitor proliferation and astrocyte differentiation (Chen et al. 2024). Interestingly, it was shown that WNT/β‐catenin may inhibit early astrogliogenesis in an Ngn2‐dependent manner (Sun et al. 2019). Our recent and former findings (Szewczyk et al. 2023) position the WNT/β‐catenin effector—TCF7L2 as an astrocytic transcription factor that plays a stage‐specific role. Downregulation of astrocyte canonical transcription factors SOX9 (Szewczyk et al. 2023) or GLI3 in *Tcf7l2* cKO mice implicates TCF7L2 involvement in astrocyte differentiation, while the downregulation of *Gja1* and *Gjb6* suggests an additional role in the final functional maturation step. At the same time, no changes in the number of cortical astrocytes in cKO mice suggest no role of TCF7L2 in regulating local cell proliferation, implicating its role in the stage and process‐specific aspects.

Our studies exclusively focus on the postnatal developmental window of astrocyte development and its effect on young adults. Hence, it only partially explains the involvement of TCF7L2 in astrocyte lineage cell development. To fully describe the stage‐specific role of TCF7L2 in astrocyte lineage cells, studies involving time and condition‐dependent deletion of *Tcf7l2* in embryonic astrocytes and progenitors are needed. This seems required and relevant since recent single‐cell RNA‐Seq data from human cortical samples during prenatal and postnatal stages of development show high expression of TCF7L2 in glial progenitors (Velmeshev et al. 2023).

We did not investigate the impact of TCF7L2 on the astrocyte post‐maturation period. The lack of such an experiment resulted from the fact that the expression level of the *Tcf7l2* gene decreases with age in cortical astrocytes, as shown by us previously. However, we cannot exclude the possibility that even a low level of TCF7L2 drives astrocyte‐neuron interaction in adulthood. Our reasoning that regulating neuronal excitability via astrocytes occurs in a cortical layer‐specific and TCF7L2‐dependent manner is based on observations only in two telencephalic structures—the cortex and hippocampus. Since astrocytes exhibit structure‐specific features and functions (Clarke et al. 2018; Khakh and Deneen 2019; Welle et al. 2021), an open question is what role TCF7L2 plays in other brain regions and structures.

Astrocytes' function can be regulated context‐specifically by other brain cells (Mallach et al. 2024). Recently, it was proposed that Wnts released by microglia during early development, in response to whisker lesioning, induce β‐catenin‐dependent transcriptional changes in astrocytes (Faust et al. 2025). TCF7L2 may act either independently or dependently on β‐catenin. Wnt molecules bind to membrane receptors and initiate a signaling cascade resulting in β‐catenin translocation to the nucleus, which binds to TCF7L2 (Bem et al. 2019). Our previous findings demonstrated nuclear localization of β‐catenin, suggesting that TCF7L2‐mediated gene expression is β‐catenin‐dependent (Szewczyk et al. 2023). Therefore, broader approaches, including different timing, structure‐specific, and context‐specific scenarios, should be considered in the future to elucidate the role TCF7L2 may play in astrocyte–neuron interactions.

In our experimental model, we observed changes in astrocyte gene expression related to ion transmembrane transport and AA transport regulation. We also documented the accumulation of glutamate, glutamine, and taurine, along with impaired potassium transport under elevated conditions, and possibly their effect on astrocyte swelling. These findings suggest the involvement of TCF7L2 as a vital factor influencing osmoregulation. In contrast to what would be expected with structural remodeling, judged by Sholl analysis, we did not observe any alterations in branching complexity. This further supports the notion that the increased astrocytic volume is more likely a consequence of cellular swelling rather than changes in overall morphological architecture. Nonetheless, we acknowledge that Sholl analysis predominantly captures the distribution and complexity of larger branches and primary processes. Therefore, to fully understand the nature of the observed changes, future analyses should aim to assess potential alterations in process motility and fine astrocytic processes, which may represent an independent factor alongside swelling in the examined model.

Astrocyte swelling has been observed in pathophysiological conditions, for example, during oxygen–glucose deprivation, hypoosmolar conditions, or elevated extracellular potassium (Murphy et al. 2017; Risher et al. 2009). AA and ionic imbalance, features of pathophysiological changes in the brain, generate osmotic pressure and change cell volume. Astrocytes respond to changed osmotic pressure, regulating their volume, for example, they swell upon an increase in glutamate concentration in cell cultures (Benesova et al. 2012; Koyama et al. 2000). This mechanism lets them increase the influx of ions and AA to reduce the extracellular level, preventing the potential cytotoxic effect of those compounds on neuronal cells. However, in our model, astrocyte‐impaired maturation caused by the loss of TCF7L2 led to changes in the expression of genes encoding AA transporters and ion channels in young adults. As a result, the astrocytic influx of AAs and ions was disrupted, and it could lead to their accumulation in the brain's ECS. This implies that in response to these pathophysiological conditions, astrocytes began to alter their cell volume—an observed effect we interpret as a consequence of the high extracellular AA level. Unfortunately, the astrocyte efforts to balance AA levels appear to be insufficient, which may directly or indirectly contribute to impaired neuronal function and excitability as suggested by correlational changes in neuronal gene expression (snRNA‐Seq) and electrophysiological properties. In this scenario, the observed effect of dysregulation of neuronal genes encoding channels and some of the SLC proteins could be the mechanism of adaptation of neurons to external conditions caused by the lack of astrocytic TCF7L2.

However, we cannot exclude the existence of mechanisms in which the *Tcf7l2*‐deficient astrocytes directly impact neuronal gene expression. Canonical transcription factors regulate neuronal genes, but the time‐ and context‐specific induction of transcription factors encoded by immediate‐early genes is one mechanism by which neurons adapt (Huang et al. 2024). Among these are orphan receptors (ORs) *Nr4a1–3*, whose expression is downregulated in cortical neurons in our model. Since astrocytes release many factors that can directly bind and stimulate ORs—such as glypican 4 (GPC4) (Condomitti et al. 2018) direct regulation of neuronal gene expression by astrocyte‐released, or in this scenario, not released molecules, represents another possible mechanism. However, further studies are needed to identify the potential astrocytic impact on ORs in cKO mice to test this hypothesis.

Our findings highlight that astrocyte dysfunction due to impaired TCF7L2 signaling leads to disrupted AA homeostasis, neuronal excitability, and gene expression. This sheds light on a previously unrecognized mechanism by which astrocytes, in a TCF7L2‐dependent manner, regulate the extracellular environment and influence neuronal function. Understanding the structure‐specific, time‐specific, and context‐dependent roles of TCF7L2 in astrocyte‐neuron interactions is needed. Our study opens new avenues for exploring TCF7L2 as a potential target in neurological disorders characterized by astrocyte dysfunction and excitability imbalance.

## Author Contributions

Conceptualization: L.M.S. and M.P. Collection and data analysis: L.M.S., M.P., J.U.‐C., K.R., T.G.K., D.A., K.G., K.H., A.Ł., and S.A.L. Investigation: L.M.S. and M.P. Visualization: L.M.S., M.P., and M.L. Funding acquisition: L.M.S. Project administration: L.M.S. Supervision: L.M.S. Writing – original draft: L.M.S. and M.P. Writing – review and editing: L.M.S. and M.P.

## Funding

This work was supported by Narodowe Centrum Nauki (National Science Centre): Sonata 16 (grant no. 2020/39/D/NZ3/03070); Opus 27 (grant no. 2024/53/B/NZ4/03058). L.M.S. was supported by Narodowe Centrum Nauki, Poland (grant 2020/39/D/NZ3/03070). M. P. was supported by Narodowe Centrum Nauki, Poland (grant no. 2023/51/D/NZ5/02035). NGS was performed thanks to Genomics Core Facility CeNT UW (RRID:SCR_022718), using the NovaSeq 6000 platform financed by the Polish Ministry of Science and Higher Education (decision no. 6817/IA/SP/2018 of 2018‐04‐10).

## Ethics Statement

All of the protocols for animal use were approved by the Polish Local Ethical Committee No. 1 in Warsaw (permission number 1227/2021). Animal use was controlled by the institutional advisory board for animal welfare at the Centre of New Technologies.

## Consent

The authors have nothing to report.

## Conflicts of Interest

Shane A. Liddelow maintains a financial interest in AstronauTx Ltd., and Synapticure and is a member of the Scientific Advisory Board of the Global BioAccess Fund. All other authors declare no conflicts of interest.

## Supporting information


**Figure S1:** Single nucleus RNA‐Seq of CTR and cKO somatosensory cortex. (A) Representative CTR (left) and cKO (right) nuclei images under light microscopy after nuclei isolation. (B) Library Traces for 20,000 nuclei capture Control 1 and cKO 1 on high‐sensitivity D1000 Screen Tape.

**Figure S2:** Identification of cell populations in CTR and cKO based on unique gene expression within cluster. (A) Average scaled expression (ASE) heatmap enriched/unique transcripts for each identified CTR (upper line) and cKO (lower line) in the identified cluster.

**Figure S3:** Scatterplots of CTR and cKO nuclei colored by maker gene expression: (A) Cluster 0, (B) Cluster 1, (C) Cluster 2, (D) Cluster 3.

**Figure S4:** Scatterplots of CTR and cKO nuclei colored by maker gene expression: (A) Cluster 4, (B) Cluster 5, (C) Cluster 6.

**Figure S5:** Scatterplots of CTR and cKO nuclei colored by maker gene expression: (A) Cluster 7, (B) Cluster 8, (C) Cluster 9, (D) Cluster 10.

**Figure S6:** Scatterplots of CTR and cKO nuclei colored by maker gene expression: (A) Cluster 11.

**Figure S7:** Dysregulation of genes involved in synaptic signaling in neurons in cKO Mice. (A) GO‐associated differentially expressed genes presented as average scaled expression (ASE) heatmaps (CTR left, cKO right) in Cluster 0, GO Term—regulation of synapse organization; (B) GO‐associated differentially expressed genes presented as average scaled expression (ASE) heatmaps (CTR left, cKO right) in Cluster 2, GO Term—chemical synaptic signaling; (C) Volcano plot of the differentially expressed genes in CTR and cKO cortical neurons, layers 4 and 5 (yellow: *p* adj. < 0.05, 0.85 > Fold Change > 1.15). (D) 10 out of 20 top Gene Ontology (GO) terms for down‐ and upregulated genes in cKO cortical neurons, layers 4 and 5. (E) GO‐associated differentially expressed genes presented as average scaled expression (ASE) heatmaps (CTR upper, cKO lower)—GO term synaptic signaling. (F) GO‐associated differentially expressed genes presented as average scaled expression (ASE) heatmaps (CTR left, cKO right) in Cluster 3, GO term—cation transmembrane transport, regulation of amino acid transport, regulation of synaptic membrane potential. (G) Violin plots of picked differentially expressed genes between CTR and cKO astrocyte clusters.

**Figure S8:** Changes in cell area in *Tcf7l2* cKO mice. (A) Quantification of the area of TdTomato+ astrocytes in the somatosensory cortex of TdT‐CTR and TdT‐cKO mice. Black and red dots represent individual astrocytes, while gray and pink dots represent individual animals; *n* = 62 astrocytes from 4 mice (TdT‐CTR) and *n* = 74 astrocytes from 4 mice (TdT‐cKO). The data were analyzed using a two‐tailed Mann–Whitney Test. (B) Quantification of the soma of TdTomato+ astrocytes in the somatosensory cortex of CTR‐TdT and cKO‐TdT mice. Black and red dots represent individual astrocytes, while gray and pink dots represent individual animals; *n* = 62 astrocytes from 4 mice (TdT‐CTR) and *n* = 74 astrocytes from 4 mice (TdT‐cKO). The data were analyzed using a two‐tailed Mann–Whitney Test (CTR‐TdT cell vs. cKO‐TdT cell) and an unpaired *t*‐test (CTR‐TdT animal vs. cKO‐TdT animal). (C) Quantification of the area of TdTomato+ astrocytes in the somatosensory cortex of AAV‐TdT CTR and AAV‐TdT cKO mice. Black and red dots represent individual astrocytes, while gray and pink represent individual animals. Calculation of the area of individual astrocytes was performed in Imaris based on three‐dimensional renders of *n* = 19 astrocytes from 4 AAV‐TdT CTR mice, *n* = 25 astrocytes from 5 AAV‐TdT cKO mice. An unpaired *t*‐test was performed to analyze group differences. (D) Quantification of the volume of GFP+ astrocytes in the somatosensory cortex of AAV‐CTR and AAV‐KO. Calculation of the area of individual astrocytes was performed in Imaris based on three‐dimensional renders of AAV‐CTR and AAV‐KO astrocytes. Black and red dots represent individual astrocytes, while gray and pink represent individual animals. Calculation of the volume of individual astrocytes was performed in Imaris based on three‐dimensional renders of *n* = 18 astrocytes from 5 AAV‐CTR mice, *n* = 21 astrocytes from 5 AAV KO mice. The data were analyzed using an unpaired *t*‐test.

**Figure S9:** Quantification of HepaCAM level in the Control and *Tcf7l2* cKO mice. (A) Quantification of *HepaCAM* gene expression in lysates of somatosensory cortex from 30‐day‐old CTR and cKO mice, using RT‐PCR, reference—*Gapdh* gene. The dot represents the mouse. (B left) Quantification of HepaCAM protein level in lysates of somatosensory cortex from 30‐day‐old CTR and cKO mice. The dot represents the mouse. (B right) Representative western blots of HepaCAM in CTR and cKO lysates.

**Figure S10:** Analysis of electrophysiological properties of hippocampal neurons in CTR and cKO mice. (A) A scheme of whole‐cell patch‐clamp recordings of hippocampal neurons from CTR and *Tcf7l2* cKO mice. (B) Resting membrane potential at −75 mV in CTR and cKO mice. The data are expressed as the mean, number of neurons: *n* = 13 neurons from 4 CTR mice; *n* = 10 neurons from 4 cKO mice. (C) A number of spikes evoked by increasing depolarizing currents at −75 mV in CTR and cKO neurons. An unpaired *t*‐test was performed to analyze group differences after assessing data distribution with the Shapiro–Wilk normality test and lognormality testing. (D) Current at maximum frequency: An unpaired *t*‐test was performed to analyze group differences after assessing data distribution with the Shapiro–Wilk normality test and lognormality testing.

**Data S1:** glia70103‐sup‐0011‐TableS1‐S8.docx.

**Data S2:** glia70103‐sup‐0012‐Supinfo.xlsx.

## Data Availability

The research data supporting this study have been deposited in the Dane Badawcze UW repository and are publicly accessible at https://doi.org/10.58132/0K8CTV, reference number 0K8CTV.
