# Supplementary figures and images for: Astrocytic TCF7L2 Impacts Brain Osmoregulation and Restricts Neuronal Excitability
Source: Glia. 2025 Dec 5;74(2):e70103. doi: 10.1002/glia.70103 (PMC12680928; doi:10.1002/glia.70103)

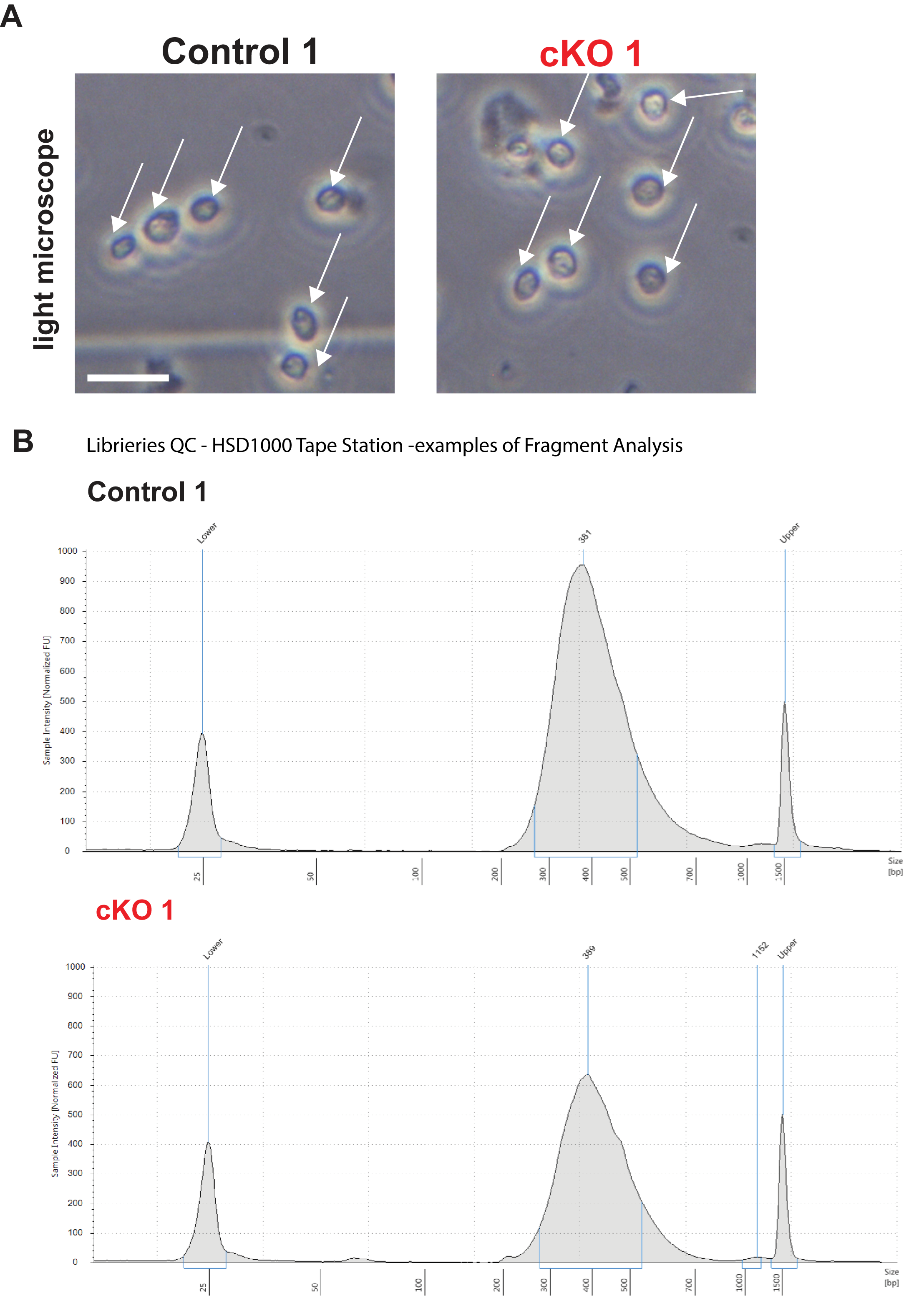

Supplement: Supplementary file 1 — Figure S1: Single nucleus RNA‐Seq of CTR and cKO somatosensory cortex. (A) Representative CTR (left) and cKO (right) nuclei images under light microscopy after nuclei isolation. (B) Library Traces for 20,000 nuclei capture Control 1 and cKO 1 on high‐sensitivity D1000 Screen Tape. [file GLIA-74-0-s012.tif]

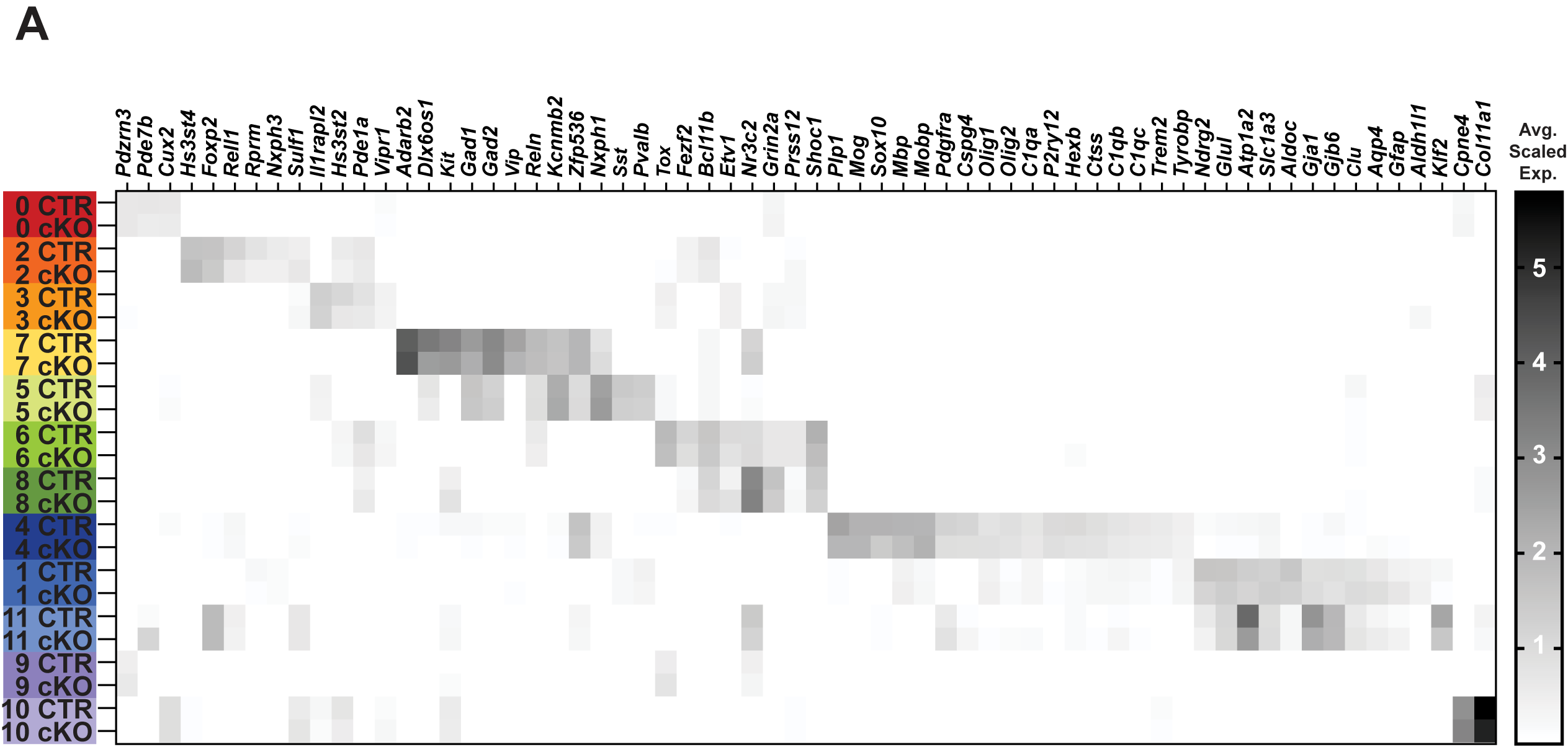

Supplement: Supplementary file 2 — Figure S2: Identification of cell populations in CTR and cKO based on unique gene expression within cluster. (A) Average scaled expression (ASE) heatmap enriched/unique transcripts for each identified CTR (upper line) and cKO (lower line) in the identified cluster. [file GLIA-74-0-s002.tif]

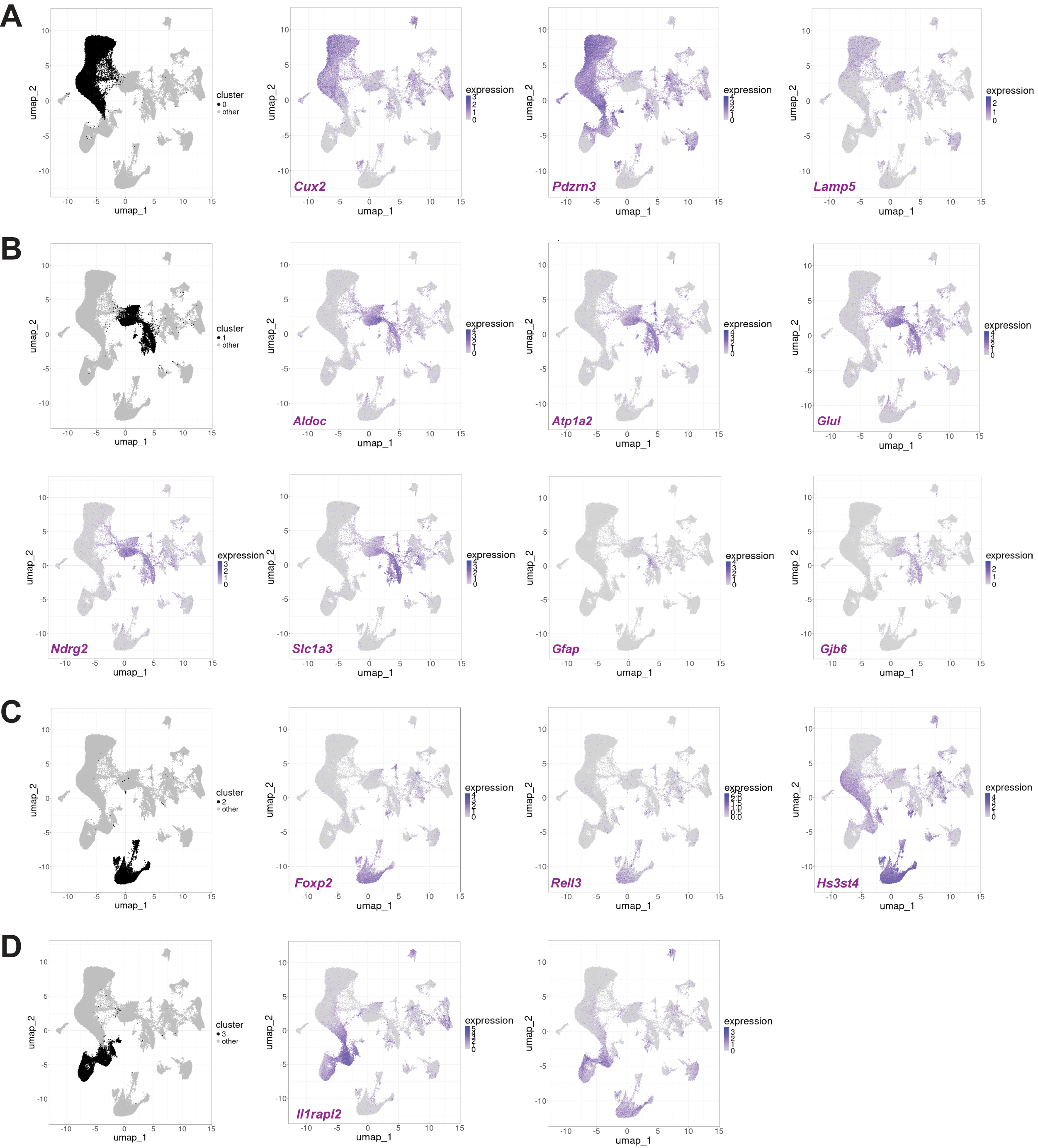

Supplement: Supplementary file 3 — Figure S3: Scatterplots of CTR and cKO nuclei colored by maker gene expression: (A) Cluster 0, (B) Cluster 1, (C) Cluster 2, (D) Cluster 3. [file GLIA-74-0-s001.tif]

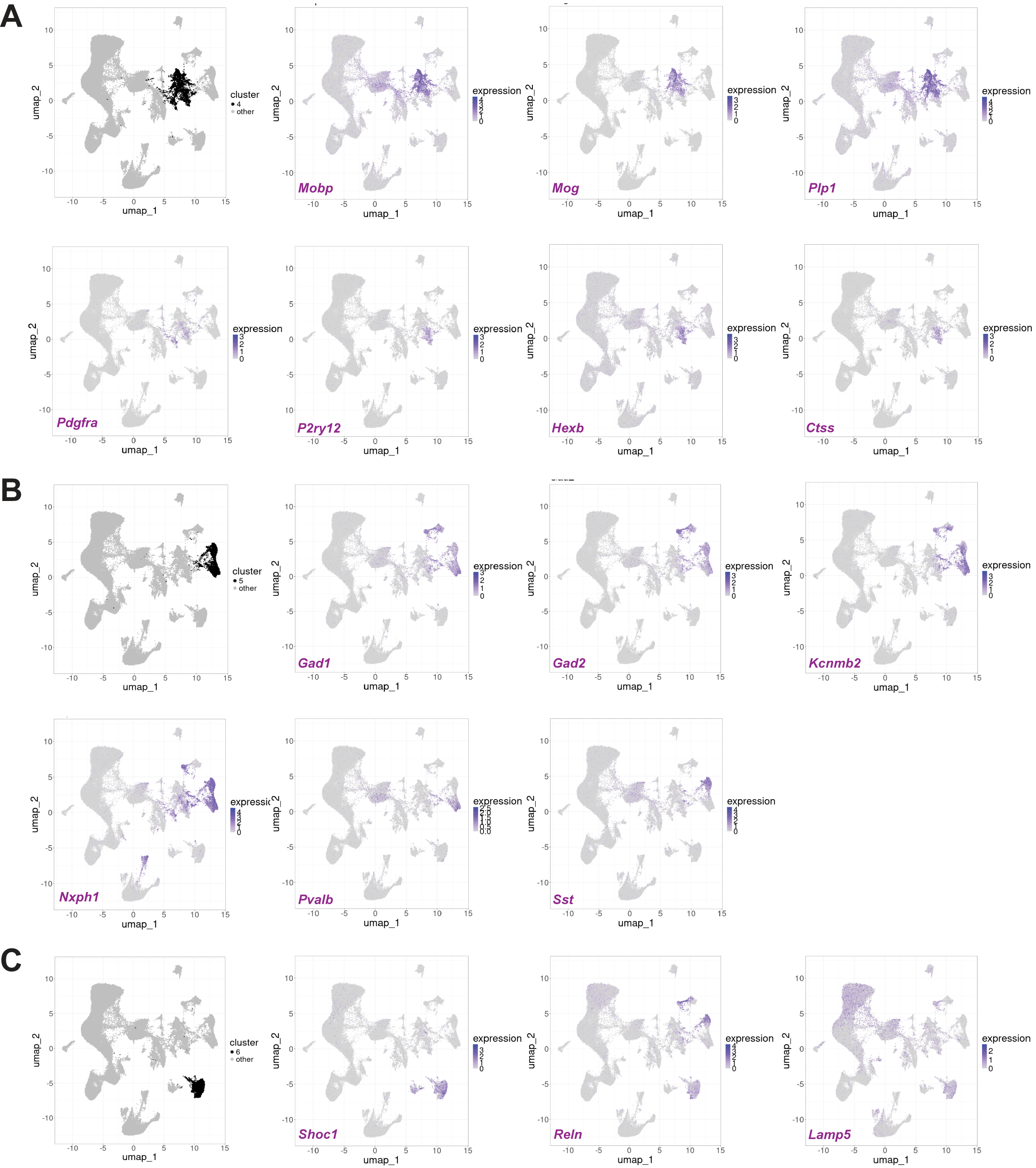

Supplement: Supplementary file 4 — Figure S4: Scatterplots of CTR and cKO nuclei colored by maker gene expression: (A) Cluster 4, (B) Cluster 5, (C) Cluster 6. [file GLIA-74-0-s011.tif]

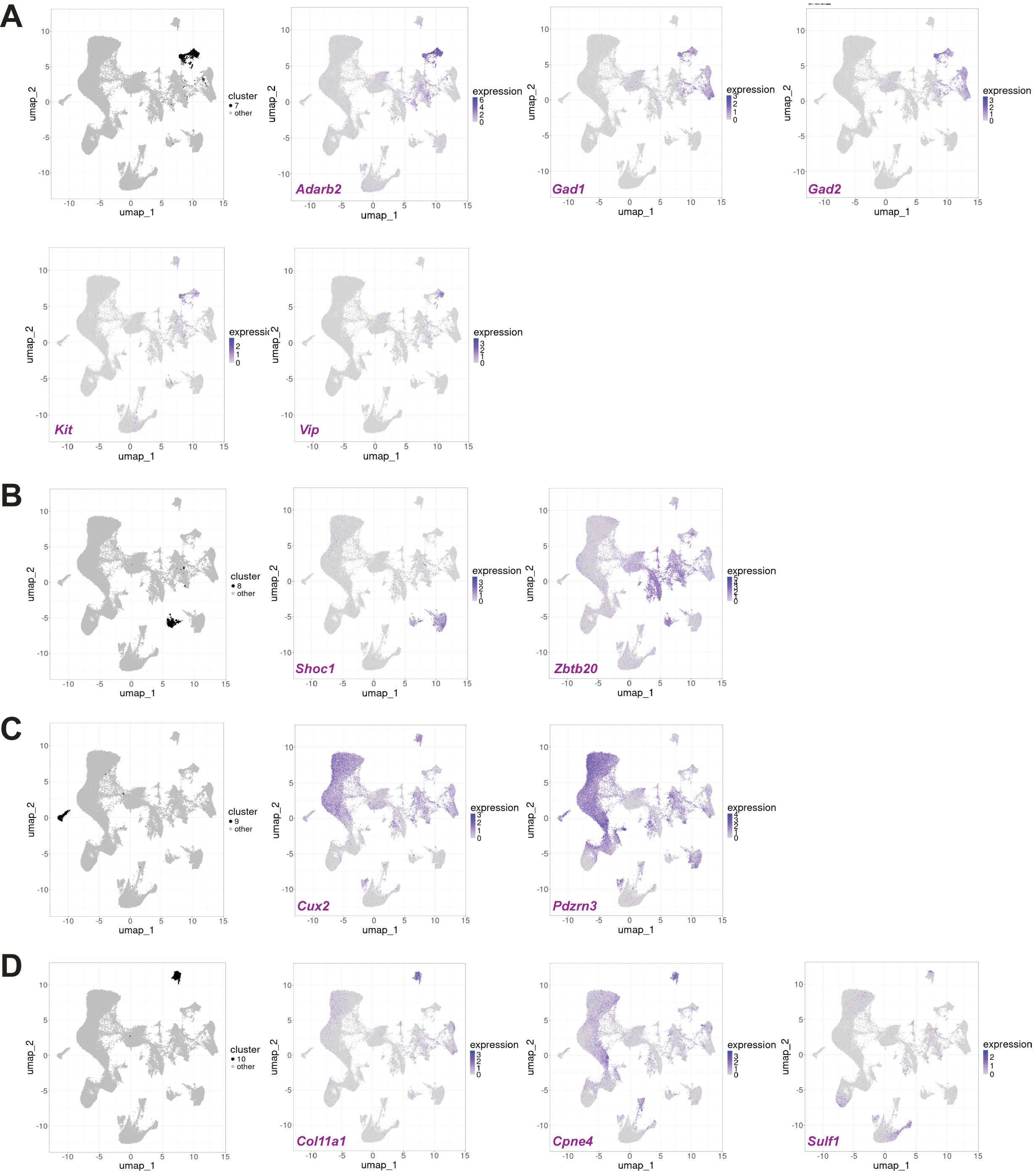

Supplement: Supplementary file 5 — Figure S5: Scatterplots of CTR and cKO nuclei colored by maker gene expression: (A) Cluster 7, (B) Cluster 8, (C) Cluster 9, (D) Cluster 10. [file GLIA-74-0-s005.tif]

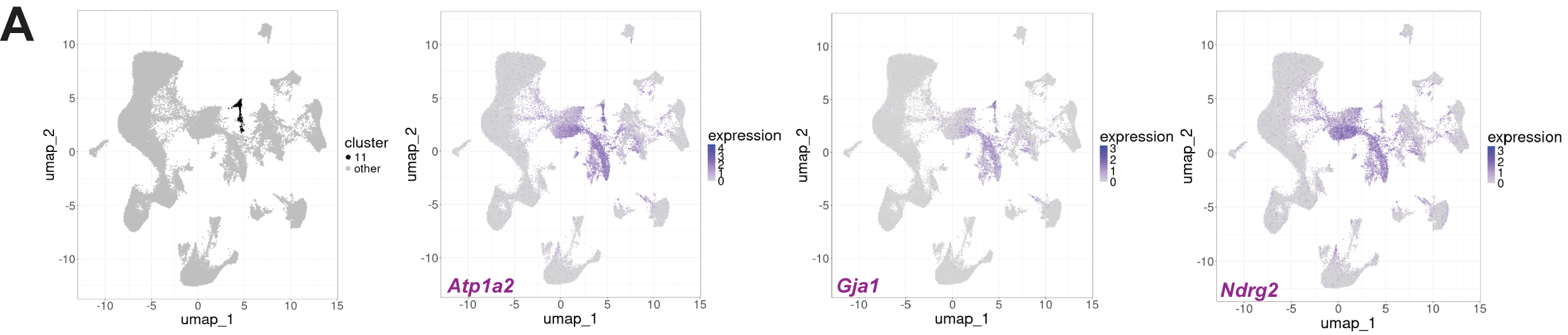

Supplement: Supplementary file 6 — Figure S6: Scatterplots of CTR and cKO nuclei colored by maker gene expression: (A) Cluster 11. [file GLIA-74-0-s006.tif]

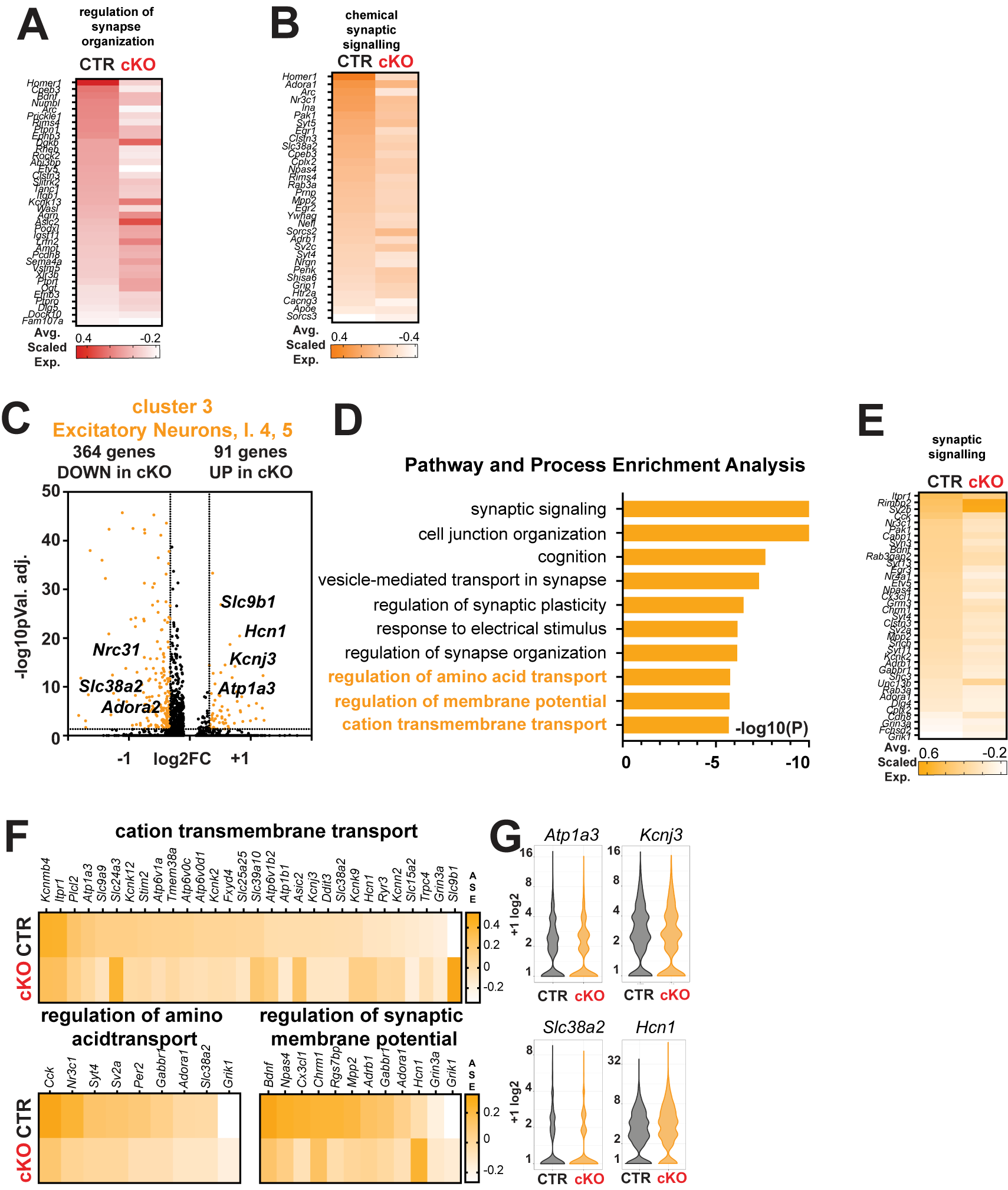

Supplement: Supplementary file 7 — Figure S7: Dysregulation of genes involved in synaptic signaling in neurons in cKO Mice. (A) GO‐associated differentially expressed genes presented as average scaled expression (ASE) heatmaps (CTR left, cKO right) in Cluster 0, GO Term—regulation of synapse organization; (B) GO‐associated differentially expressed genes presented as average scaled expression (ASE) heatmaps (CTR left, cKO right) in Cluster 2, GO Term—chemical synaptic signaling; (C) Volcano plot of the differentially expressed genes in CTR and cKO cortical neurons, layers 4 and 5 (yellow: p adj. < 0.05, 0.85 > Fold Change > 1.15). (D) 10 out of 20 top Gene Ontology (GO) terms for down‐ and upregulated genes in cKO cortical neurons, layers 4 and 5. (E) GO‐associated differentially expressed genes presented as average scaled expression (ASE) heatmaps (CTR upper, cKO lower)—GO term synaptic signaling. (F) GO‐associated differentially expressed genes presented as average scaled expression (ASE) heatmaps (CTR left, cKO right) in Cluster 3, GO term—cation transmembrane transport, regulation of amino acid transport, regulation of synaptic membrane potential. (G) Violin plots of picked differentially expressed genes between CTR and cKO astrocyte clusters. [file GLIA-74-0-s008.tif]

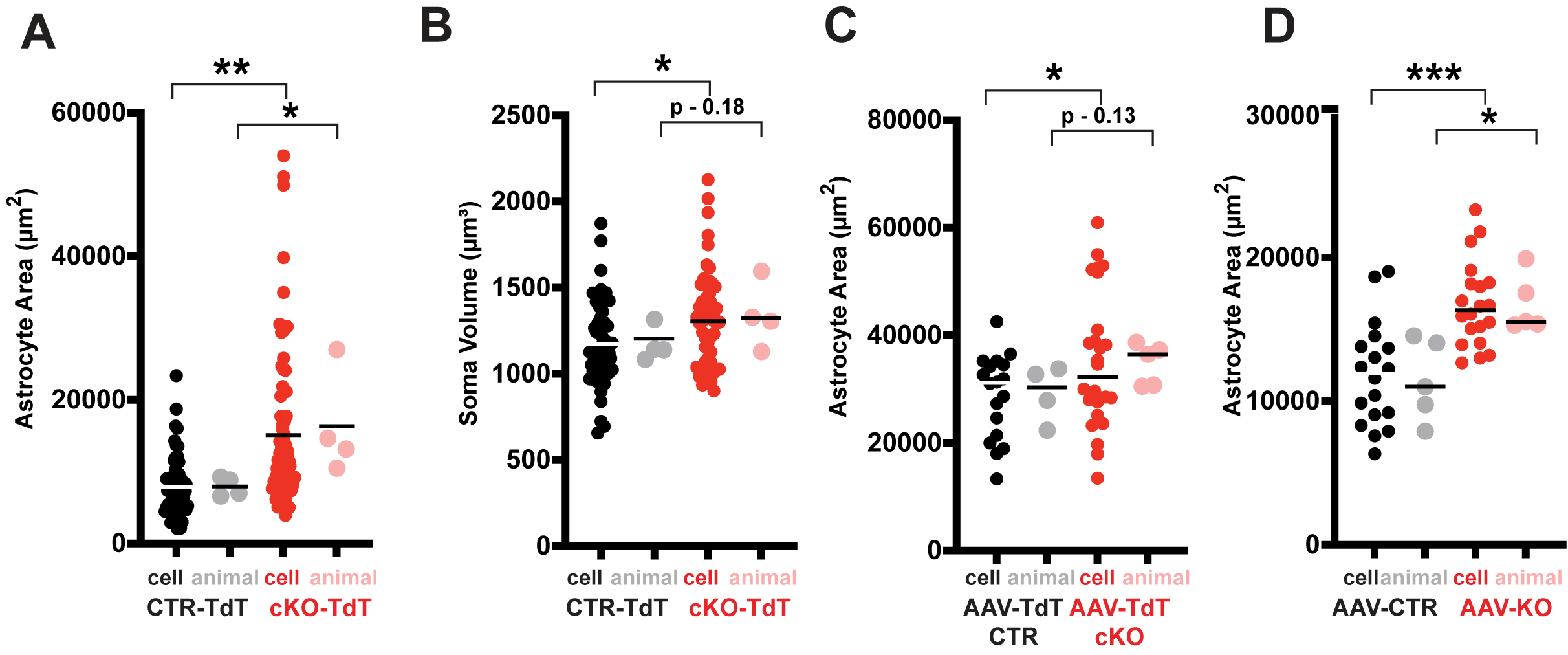

Supplement: Supplementary file 8 — Figure S8: Changes in cell area in Tcf7l2 cKO mice. (A) Quantification of the area of TdTomato+ astrocytes in the somatosensory cortex of TdT‐CTR and TdT‐cKO mice. Black and red dots represent individual astrocytes, while gray and pink dots represent individual animals; n = 62 astrocytes from 4 mice (TdT‐CTR) and n = 74 astrocytes from 4 mice (TdT‐cKO). The data were analyzed using a two‐tailed Mann–Whitney Test. (B) Quantification of the soma of TdTomato+ astrocytes in the somatosensory cortex of CTR‐TdT and cKO‐TdT mice. Black and red dots represent individual astrocytes, while gray and pink dots represent individual animals; n = 62 astrocytes from 4 mice (TdT‐CTR) and n = 74 astrocytes from 4 mice (TdT‐cKO). The data were analyzed using a two‐tailed Mann–Whitney Test (CTR‐TdT cell vs. cKO‐TdT cell) and an unpaired t‐test (CTR‐TdT animal vs. cKO‐TdT animal). (C) Quantification of the area of TdTomato+ astrocytes in the somatosensory cortex of AAV‐TdT CTR and AAV‐TdT cKO mice. Black and red dots represent individual astrocytes, while gray and pink represent individual animals. Calculation of the area of individual astrocytes was performed in Imaris based on three‐dimensional renders of n = 19 astrocytes from 4 AAV‐TdT CTR mice, n = 25 astrocytes from 5 AAV‐TdT cKO mice. An unpaired t‐test was performed to analyze group differences. (D) Quantification of the volume of GFP+ astrocytes in the somatosensory cortex of AAV‐CTR and AAV‐KO. Calculation of the area of individual astrocytes was performed in Imaris based on three‐dimensional renders of AAV‐CTR and AAV‐KO astrocytes. Black and red dots represent individual astrocytes, while gray and pink represent individual animals. Calculation of the volume of individual astrocytes was performed in Imaris based on three‐dimensional renders of n = 18 astrocytes from 5 AAV‐CTR mice, n = 21 astrocytes from 5 AAV KO mice. The data were analyzed using an unpaired t‐test. [file GLIA-74-0-s010.tif]

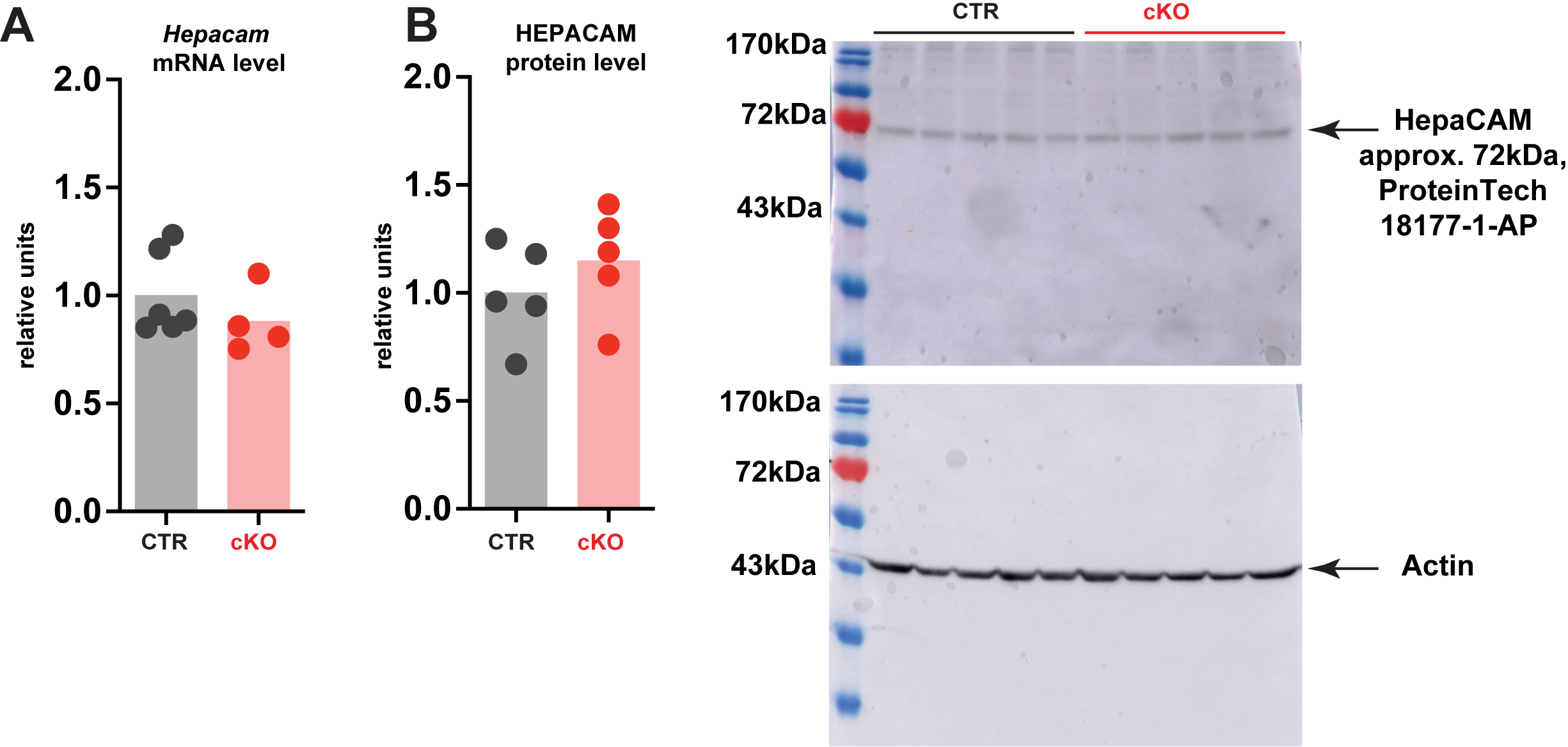

Supplement: Supplementary file 9 — Figure S9: Quantification of HepaCAM level in the Control and Tcf7l2 cKO mice. (A) Quantification of HepaCAM gene expression in lysates of somatosensory cortex from 30‐day‐old CTR and cKO mice, using RT‐PCR, reference—Gapdh gene. The dot represents the mouse. (B left) Quantification of HepaCAM protein level in lysates of somatosensory cortex from 30‐day‐old CTR and cKO mice. The dot represents the mouse. (B right) Representative western blots of HepaCAM in CTR and cKO lysates. [file GLIA-74-0-s004.tif]

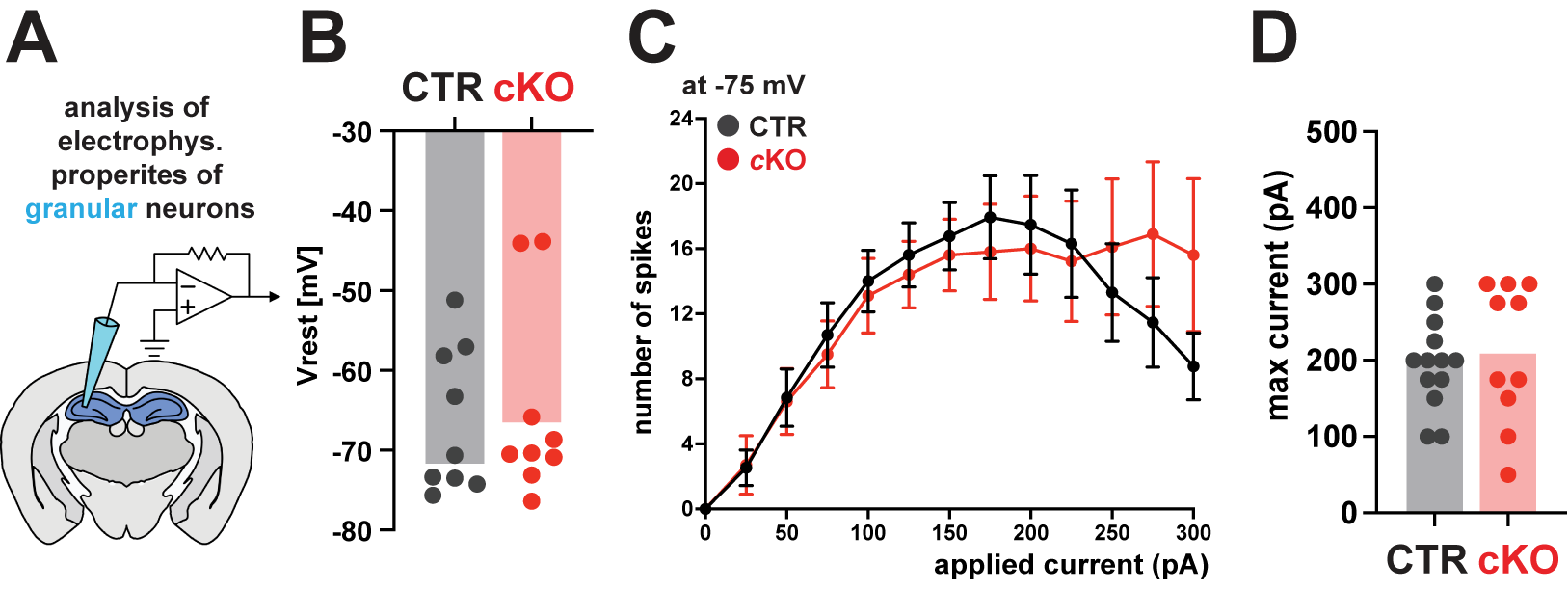

Supplement: Supplementary file 10 — Figure S10: Analysis of electrophysiological properties of hippocampal neurons in CTR and cKO mice. (A) A scheme of whole‐cell patch‐clamp recordings of hippocampal neurons from CTR and Tcf7l2 cKO mice. (B) Resting membrane potential at −75 mV in CTR and cKO mice. The data are expressed as the mean, number of neurons: n = 13 neurons from 4 CTR mice; n = 10 neurons from 4 cKO mice. (C) A number of spikes evoked by increasing depolarizing currents at −75 mV in CTR and cKO neurons. An unpaired t‐test was performed to analyze group differences after assessing data distribution with the Shapiro–Wilk normality test and lognormality testing. (D) Current at maximum frequency: An unpaired t‐test was performed to analyze group differences after assessing data distribution with the Shapiro–Wilk normality test and lognormality testing. [file GLIA-74-0-s003.tif]
