# Supplementary material for: Astrocytic TCF7L2 Impacts Brain Osmoregulation and Restricts Neuronal Excitability
Source: Glia. 2025 Dec 5;74(2):e70103. doi: 10.1002/glia.70103 (PMC12680928; doi:10.1002/glia.70103)
Supplement: Supplementary file 11 — Data S1: glia70103‐sup‐0011‐TableS1‐S8.docx. [file GLIA-74-0-s007.docx]

**Lists of mouse strains, plasmids, antibodies, assays, kits, software, and chemical compounds used in studies containing sources and identifiers:**

**Table 1: List of mouse strains used in the studies**

| **Mice line** | SOURCE | IDENTIFIER |
| --- | --- | --- |
| C57BL/6NTac-Tcf7l2^tm1a^(EUCOMM)Wtsi/WtsiIeg | EUCOMM | EM:07858  RRID:IMSR_EM:07858 |
| B6;FVB-Tg(Aldh1l1-cre/ERT2)1Khakh/J | Jackson Labs | Strain #:029655  RRID:IMSR_JAX:029655 |
| B6.Cg-*Gt(ROSA)26Sor^tm9(CAG-tdTomato)Hze^*/J | Jackson Labs | Strain #:007909  RRID:IMSR_JAX:007909 |
| B6J.129(B6N)-Gt(ROSA)26Sortm1(CAG-cas9*-EGFP)Fezh/J | Jackson Labs | Strain #:026175  RRID:IMSR_JAX:026175 |
| B6.129S4-Gt(ROSA)26Sortm1(FLP1)Dym/RainJ | Jackson Labs | Strain #:009086  RRID:IMSR_JAX:009086 |

**Table 2: List of plasmids used in the studies**

| **Plasmid name** | SOURCE | IDENTIFIER |
| --- | --- | --- |
| pZac2.1gfaABC1D-tdTomato | Addgene | RRID:Addgene_44332  44332 |
| AAV:ITR-U6-sgRNA(backbone)-hSyn-Cre-2A-EGFP-KASH-WPRE-shortPA-ITR | Addgene | RRID:Addgene_60231  60231 |
| pAAV:ITR-U6-sgRNA(anti-*lacZ*)-gfaABC1D-Cre (control) | this study | --- |
| pAAV:ITR-U6-sgRNA-(anti-*Tcf7l2*)-gfaABC1D-Cre (*Tcf7l2* KO) | this study | --- |

**Table 3: List of Antibodies used in studies**

| **Antibodies** | SOURCE | IDENTIFIER |
| --- | --- | --- |
| Anti-HepaCAM | ProteinTech | 18177-1-AP |
| Anti-EGFP | Abcam | ab13970 |
| anti-rabbit IgG peroxidase antibody, Sigma Aldrich, | Merck | A0545 |
| Goat anti-Chicken IgY (H+L) Secondary Antibody, Alexa Fluor™ 488 | Thermo Fisher | A-11039 |

**Table 4: List of critical commercial assays used in studies**

| **Assays** | SOURCE | IDENTIFIER |
| --- | --- | --- |
| PIPSeq^TM^ T20 3” Single Cell RNA Kit v4.0 | Fluent BioSciences, US | FBS-SCR-T20-4- V4 |
| Nuclei Isolation Kit: Nuclei EZ Prep | Merck | NUC101-1KT |
| SYBR Green I Master Kit | Roche | 04707516001 |
|  |  |  |

**Table 5: List of chemical compounds used in studies**

| **Chemical compounds** | SOURCE | IDENTIFIER |
| --- | --- | --- |
| Protector RNase Inhibitor | Merck | 3335399001 |
| Bovine Serum Albumin | Merck | 98806-5G |
| DPBS | Thermo Scientific | 14040174 |
| ketamine | Biowet | 101788 |
| xylazine | Biowet | SEDAZIN 20 |
| PFA | Merck | P6148 |
| sucrose | Merck | 1076515000 |
| O.C.T | Sakura Tissue-Tek, | 4583 |
| isopentane | VWR | 24872.298 |
| glycerol | VWR | 443320113 |
| citrate buffer | Bioshop Life Science Products | CIT001.1 |
| Tween20 | VWR | 663684B |
| Fluoromount G | Thermo Scientific | 00-4959-52 |
| Benzonase Nuclease | Merck | 70746 |
| Tris | Bioshop Life Science Products | TRS001.1 |
| NaCl, | Chempur | 794121116 |
| NP40 | Thermo Scientific | 85124 |
| sodium deoxycholate | Merck | D6750 |
| sodium dodecyl sulfate | Bioshop Life Science Products | SDS999.500 |
| EDTA | VWR | E177-500ML |
| NaF | Merck | 67414-1ML-F |
| cOmplete, EDTA-free Protease Inhibitor | Roche | 4693132001 |
| phosphatase inhibitor | Roche | 4906845001 |
| poly-acrylamide gels | Biorad | 1610183 |
| nitrocellulose membranes | Biorad | 1620112 |
| cOmplete, EDTA-free Protease Inhibitor | Roche | 4693132001 |
| phosphatase inhibitor | Roche | 4906845001 |
| poly-acrylamide gels | Biorad | 1610183 |
| nitrocellulose membranes | Biorad | 1620112 |
| DNase | ThermoFisher Scientific | AM2238 |
| SmaI | ThermoFisher | FD0663 |
| Betadine | EGIS Pharmaceuticals | 18509 |
| Butomidor | Orion Pharma Poland Sp. z o.o. | 0520462AD |
| Iso-Vet | Piramal Critical Care B. V. | IN/EL/0035/19/01 |
| Ophthalmic gel | Vidisic | --- |
| NaCl | Sigma-Aldrich | S9888 |
| KCl | Chempur | 117397402 |
| CaCl2 | Sigma-Aldrich | 499609 |
| MgSO4 | Sigma-Aldrich | M7506 |
| KH2PO4 | Merck | 5.43841 |
| K2HPO4 | Merck | 5.43839 |
| NaHCO3 | Chempur | 118105307 |
| D-glucose | Chempur | 114595600 |
| Phthaldialdehyde | Merck | 1.11452 |
| 2-Mercaptoethanol | Sigma-Aldrich | M6259 |
| Methanol | Merck | 1.06007 |
| Acetonitrile Chromasolv | Sigma-Aldrich | 34851 |

**Table 6: List of software and algorithms used in research**

| **Software and algorithms** | SOURCE | IDENTIFIER |
| --- | --- | --- |
| Imaris | Bitplane | RRID:SCR_007370 |
| Graphpad prism | Prism | RRID:SCR_002798 |
| Quantity One 1‐D software | Biorad | **RRID:SCR_014280** |
| Seurat | open-source | RRID:SCR_016341 |
| Chromeleon™ Chromatography Data System (CDS) Software | Thermo Scientific™ | 6.80 SR13 |

**Table 7: List of deposited data information**

| **Deposited data identifier** | SOURCE | IDENTIFIER |
| --- | --- | --- |
| Single nucleus RNA-Seq Data | Geo | GSE295953 |
|  |  |  |

**Table 8: List of other reagents and resources used in studies**

| **Other reagents ans resources** | SOURCE | IDENTIFIER |
| --- | --- | --- |
| Confocal Microscope | Zeiss | Axio Imager Z2 LSM 700 |
| Kimble Dounce tissue grinder set | Merck | D8938 |
| LightCycler 480 Instrument II | Roche | 05015278001 |
| ImageQuant LAS 4000 | Cytiva | LAS 4000 |
| Stereotaxic Apparatus | Neurostar | SD46 |
| Nanofil syringe | World Precision Instruments | 09J |
| 40 um cell strainer | Corning Life Science | 352340 |
| 10 um cell strainer | pluriSelect Life Science | 43-10010-40 |
| Atomic emission spectrometer (ICP-AES) | Thermo Scientific iCAP 7400 | iCAP 7400 |
| Cryostat | Leica | CM1860 |
| HiTrap Heparin HP Column | Cytiva | 17040601 |
| Amicon Ultra-4 Centrifugal Filter NMWL 100 KDa | Millipore | UFC810024 |
| Nanosep MF 0.2 µm centrifugal filter | Pall, | ODPTFE02C34 |
| NovaSeq | Illumina | 6000 |
| TapeStation | Agilent | 4200 |
| Qubit 4 | Thermo Fisher | --- |
| SYBR Green I Master Kit | Roche | 04707516001 |
| Matrix VIP 3000 Isoflurane Vaporizer | MIDMARK | V1439867 |
| Occulus Quest | Meta |  |
| CMA 110 Liquid Switch | CMA Microdialysis | 8308200 |
| CMA 7 6 kDa Microdialysis Probe, 1 mm membrane length | CMA Microdialysis | P000082 |
| Hypersil GOLD™ C18 Selectivity HPLC Column | Thermo Fisher | 25005-254630 |
| Thermo Scientific UltiMate 3000 HPLC System (Pump, Autosampler, Column Compartment) | Thermo Fisher | --- |
| RF 2000 Fluorescence Detector | Thermo Scientific™ | RF 2000 |
| Digital Lab Standard Stereotaxic | Stoelting co. | 51500D |
